# The Stimulating Effect of Low-Molecular-Weight Luteinizing Hormone Receptor Agonist on Steroidogenesis and Ovulation in Female Rats with Dehydroepiandrosterone-Induced Polycystic Ovary Syndrome

**DOI:** 10.3390/ijms27062748

**Published:** 2026-03-18

**Authors:** Kira V. Derkach, Alena S. Pechalnova, Inna I. Zorina, Irina Yu. Morina, Liubov V. Bayunova, Irina V. Romanova, Irina V. Fedorchuk, Julian R. Ryzhov, Elizaveta E. Chernenko, Viktor N. Sorokoumov, Alexander O. Shpakov

**Affiliations:** Laboratory of Molecular Endocrinology and Neurochemistry, Sechenov Institute of Evolutionary Physiology and Biochemistry of Russian Academy of Sciences, 194223 Saint Petersburg, Russia; derkatch_k@list.ru (K.V.D.); pechalnova.alena@gmail.com (A.S.P.); zorina.inna.spb@gmail.com (I.I.Z.); irinamorina@mail.ru (I.Y.M.); bayunoval@mail.ru (L.V.B.); irinaromanova@mail.ru (I.V.R.); fedorchuk1969@mail.ru (I.V.F.); julian.ryzhov@gmail.com (J.R.R.); llystdi@gmail.com (E.E.C.); sorokoumov@gmail.com (V.N.S.)

**Keywords:** polycystic ovary syndrome, luteinizing hormone/chorionic gonadotropin receptor, progesterone, ovulation, ovarian steroidogenesis, allosteric agonist, dehydroepiandrosterone, thieno[2,3-d]-pyrimidine

## Abstract

Polycystic ovary syndrome (PCOS) is associated with impaired ovarian steroidogenesis and ovulation, which necessitates the development of effective ovulation inducers for PCOS. The aim of the study was to evaluate the effects of allosteric luteinizing hormone receptor agonist TP03 and human chorionic gonadotropin (hCG) on ovarian steroidogenesis, as well as ovulation in prepubertal female rats with dehydroepiandrosterone(DHEA)-induced PCOS. Taking into account differences in progesterone levels, cohorts with high (PCOS(H)) and low (PCOS(L)) progesterone were formed and treated with Follimag and Cetrotide. After 48 h, TP03 (25 mg/kg) or hCG (25 IU/rat) were injected, and hormone levels, gene expression, and ovarian morphology were assessed. The PCOS(H)-cohort exhibited irregular estrous cycles, ovarian cysts, and increased ovarian mass and estradiol levels, but the number of corpora lutea (CL) was maintained. In the PCOS(L)-cohort, ovarian weight was increased, and *Star*, *Cyp11a1*, and *Adamts1* gene expression as well as the CL number were decreased. In both cohorts, TP03 and hCG increased progesterone levels and the expression of steroidogenesis (*Star*, *Cyp11a1*) and ovulation (*Cox2*, *Adamts1*, *Egr1*) genes, as well as inducing CL formation. Thus, TP03, like hCG, stimulates steroidogenesis and ovulation in PCOS-rats with different progesterone levels, which provides the first evidence of the effectiveness of allosteric LHR agonists as ovulation triggers in PCOS.

## 1. Introduction

Polycystic ovary syndrome (PCOS) is one of the most common endocrine disorders in women, most often developing in the prepubertal period [1,2,3]. Its prevalence depends on the studied population, its age, and ethnic composition. Depending on the selected assessment criteria, the incidence of PCOS ranges from 6% to 13% of women [2,4,5]. According to the Rotterdam criteria, PCOS is diagnosed based on the presence of at least two features in the patient: clinical or biochemical hyperandrogenism, ovulatory dysfunction, and polycystic ovary morphology or elevated anti-Müllerian hormone levels [5,6,7,8], with hyperandrogenism being the leading factor in the etiology and pathogenesis of PCOS [9,10,11,12,13].

Given this etiology, animal models of PCOS are therefore designed to mimic the condition by exposing the ovaries to androgen excess [14,15]. The most widely used models of PCOS are those induced by long-term treatment of rodents with dehydroepiandrosterone (DHEA), a testosterone precursor [14], with models induced by prepubertal DHEA treatment of rats being the closest to the disease in humans [16,17].

One of the key problems in controlled ovarian stimulation in patients with PCOS is the impaired ovarian response to the stimulating effect of gonadotropins, which can result in both the development of ovarian hyperstimulation syndrome (OHSS), which poses a serious risk to a woman’s health, and a decrease in the number of obtained mature oocytes [18]. Reduced endometrial receptivity and disturbances in the early stages of embryo development also pose a significant problem when performing assisted reproductive technologies (ARTs) in patients with PCOS, leading to a decrease in the clinical pregnancy rate [5,18,19]. The development and implementation of new in vitro fertilization protocols for patients with PCOS, including the use of gonadotropin-releasing hormone (GnRH) antagonists [7] and progestins [20,21], reduces the risk of OHSS but does not significantly increase the live birth rate [7,22]. This necessitates the development of new pharmacological approaches to controlled ovulation stimulation in this endocrine disorder. Of greatest interest among them are various agonists of the luteinizing hormone receptor (LHR), both gonadotropin analogs with luteinizing hormone (LH) activity and low-molecular-weight allosteric agonists of LHR [23,24,25].

Therefore, evaluating the effects of LHR agonists on ovarian steroidogenesis, ovulation, and fertility in PCOS rodent models is a necessary step in developing these therapeutic strategies. However, data on the stimulating effect of LH-active gonadotropins on ovulation in PCOS are scarce and contradictory, and there are no data on DHEA-induced PCOS. The effects of low-molecular-weight allosteric LHR agonists on ovarian steroidogenesis and ovulation in PCOS have not been previously studied. Simultaneously, studies have shown that partial LHR agonists, such as the Dutch-developed Org43553 [26] and our compounds TP03 and TP4/2 [27,28], stimulate ovarian steroidogenesis and ovulation in female rats [29,30]. Notably, in female volunteers, Org43553 also stimulates ovulation [31]. It is important that all these compounds, which belong to the thieno[2,3-d]-pyrimidine derivatives, do not interfere with the activation of LHR by endogenous gonadotropins [25,26,27], do not cause hyperactivation of LHR due to being one of the primary causes of OHSS [29,30], and do not lead to desensitization of LHR during course administration [27,28].

In accordance with the above, the aim of the study was to evaluate the effect of the previously developed allosteric agonist 5-amino-*N-tert*-butyl-2-(methylsulfanyl)-4-(3-(nicotinamido)phenyl)thieno[2,3-d]-pyrimidine-6-carboxamide (TP03) on ovarian steroidogenesis and ovulation in prepubertal female rats with DHEA-induced PCOS. This study was carried out in comparison with hCG, the “gold standard” of ovulation inducers, which is important both for assessing the efficacy of LHR agonists with different mechanisms of action in PCOS and for optimizing the use of gonadotropins in the in vitro fertilization protocols in patients with PCOS. Since the DHEA model of PCOS in prepubertal female rats, despite a number of potential advantages over the “early” DHEA model of this disease [16,17], has been insufficiently studied, we characterized it at the initial stage of the work based on morphological, metabolic, and hormonal parameters.

## 2. Results

### 2.1. Characteristics of Female Rats with DHEA-Induced PCOS by Body Weight, Total and Specific Ovarian Mass, and Hormonal and Metabolic Status

**Figure 1:**
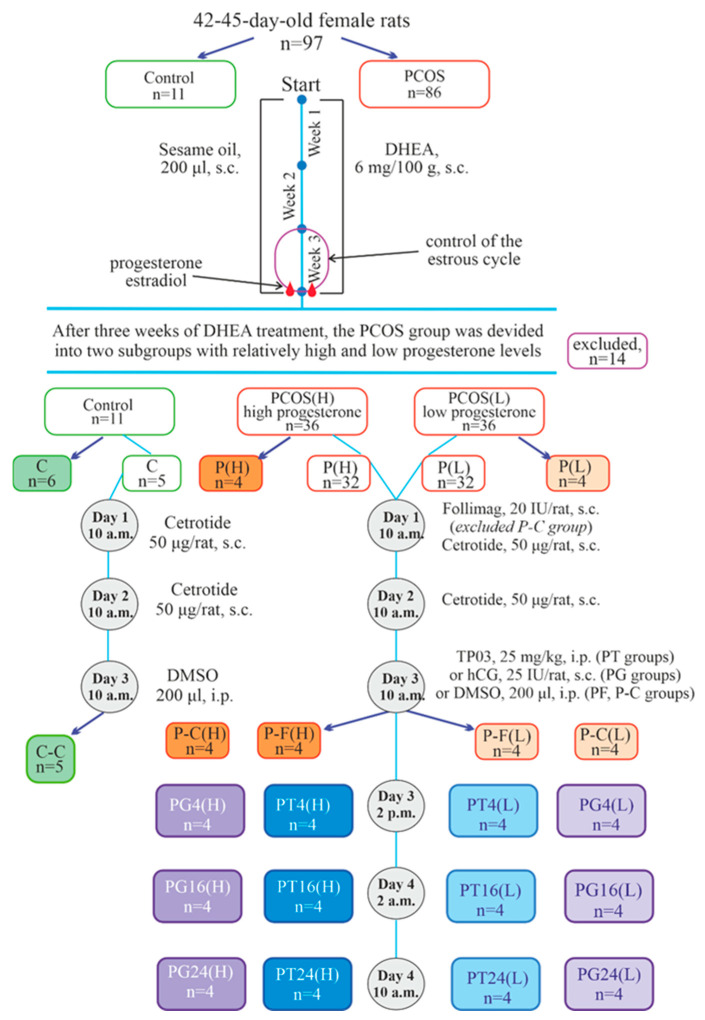
Experimental design and groups of control and PCOS animals. To induce PCOS, 42–45-day-old female rats (*n* = 86) were treated with DHEA (6 mg/100 g/day) for 3 weeks. Control rats of the same age (*n* = 11) received sesame oil instead of DHEA. During the last 10 days of DHEA treatment, vaginal smears were taken to assess the phases of the estrous cycle, and rats with an irregular cycle were considered as PCOS animals (72 rats). At the end of the third week, progesterone and estradiol levels were assessed, and the animals were divided into cohorts, with progesterone levels above (PCOS(H)) and below (PCOS(L)) being the median value (36 animals in each). Each cohort was randomly divided into 9 groups (4 rats in each). Two PCOS groups from the cohorts with high (P(H), *n* = 4)- and low (P(L), *n* = 4)-progesterone were used to characterize the PCOS model, and they were compared with control rats treated with sesame oil (C, *n* = 6). Two more groups from the high- (P-C(H), *n* = 4) and low (P-C(L), *n* = 4) progesterone cohorts were treated twice with Cetrotide (50 μg/rat per injection, s.c.) at a 24 h interval, and their ovarian morphology was assessed and compared with control rats treated with Cetrotide (C-C, *n* = 5). The remaining PCOS rats were treated with Follimag (20 IU/rat, s.c.) and twice with Cetrotide. Forty-eight hours after Follimag treatment, the PCOS rats were given a single injection of TP03 (25 mg/kg, three groups from each cohort) or hCG (25 IU/rat, three groups from each cohort) or 200 μL of DMSO (one group from each cohort). PCOS rats treated with TP03 or hCG were euthanized 4, 16, and 24 h after administration of LHR agonists. The following groups of PCOS rats were formed (*n* = 4 in each): with DMSO—P-F(H) and P-F(L); with TP03 and euthanasia after 4, 16, and 24 h—PT4(H), PT4(L), PT16(H), PT16(L), PT24(H), and PT24(L); with hCG and euthanasia after 4, 16, and 24 h—PG4(H), PG4(L), PG16(H), PG16(L), PG24(H), and PG24(L).

PCOS was induced in 42–45-day-old female rats by subcutaneous injections of DHEA (6 mg/100 g body weight/day) dissolved in sesame oil for 3 weeks. Age-matched control rats received vehicle (sesame oil) only. The design of the experiment is shown in Figure 1.

**Figure 2:**
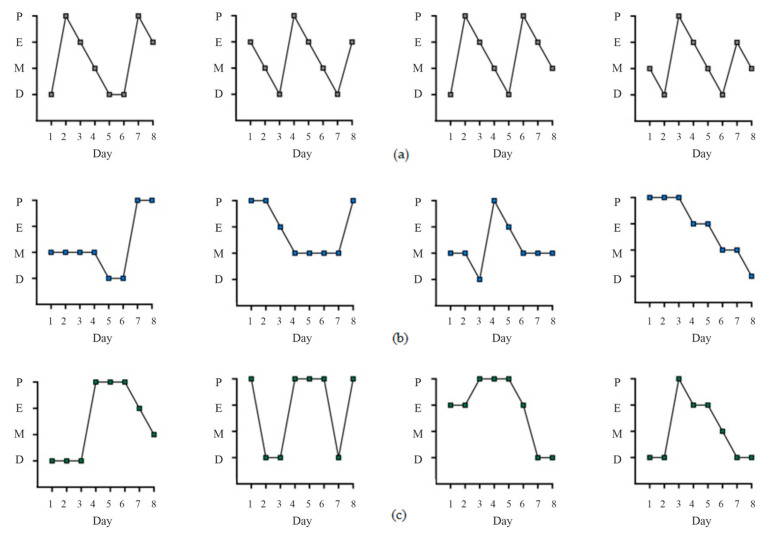
Estrous cycles in control rats (**a**) and in PCOS rats with high (**b**) and low (**c**) progesterone levels in the blood. Estrous cycle phases were assessed over an 8-day period. Designations: P—proestrus, E—estrus, M—metestrus, D—diestrus.

During the final 10 days of DHEA treatment, vaginal smears were taken from the animals, and the estrous cycle phases were assessed, selecting rats with irregular cycles. Among the DHEA-treated female rats, pronounced estrous cycle disturbances indicative of PCOS development were demonstrated in 72 female rats (84% of the total number (*n* = 86) of DHEA-treated animals) (Figure 2). Blood progesterone levels were assessed to divide them into two cohorts with high (PCOS(H)) and low (PCOS(L)) hormone concentrations (see Section 4 for details) (Table 1). Progesterone levels in the PCOS(H) cohort differed significantly from those in the control, while progesterone levels in the PCOS(L) cohort did not differ from those in the control groups. Unlike progesterone, body weight and blood estradiol levels in PCOS rats showed a relatively narrow spread (Table 1). However, when comparing estradiol levels in the PCOS(H) and PCOS(L) cohorts, an increase in blood estradiol concentrations was shown in the PCOS(H) cohort compared to both controls and PCOS(L) (Table 1). The nature of estrous cycle disturbances in PCOS rats with high and low progesterone levels also differed, particularly in the metestrus and diestrus phases. In the PCOS(H) cohort, animals were more often in the metestrus phase, while in the PCOS(L) cohort, they were more often in the diestrus and proestrus phases (Figure 2).

Morphometric, metabolic, and hormonal parameters were then studied in the P(H) and P(L) groups of PCOS rats, not treated with Follimag or ovulation inducers. Body weight in these groups did not differ from that in the control. Compared with the control animals, the P(H) group had increased ovarian weight, its ratio to body weight, and levels of progesterone, estradiol, and testosterone, while the P(L) group showed an increase in the specific weight of the ovaries and testosterone level (Table 2). There were significant differences in blood progesterone and estradiol levels between the P(H) and P(L) groups. The LH level in the blood of both groups of PCOS rats was reduced to the same extent (Table 2).

When comparing the control and both groups of PCOS rats, no differences were found in fasting glucose levels and those 120 min after the glucose load, AUC_0–120_ values, integrated area under the glucose curve in the intraperitoneal glucose tolerance test (iGTT), leptin levels before and after the glucose load, basal insulin levels, and the IR0 index calculated from them (Table 2). At the same time, in the P(H) group (but not in the P(L) group), a tendency toward an increase in the insulin level 120 min after the glucose load (*p* = 0.056 compared to C) and a significant increase in the IR120 index were observed (Table 2). These data indicate the absence of noticeable metabolic disorders in DHEA-induced PCOS rats, with the exception of initial signs of IR development in rats from PCOS(H) cohort.

### 2.2. Evaluation of Ovarian Gene Expression in Female Rats with DHEA-Induced PCOS

**Figure 3:**
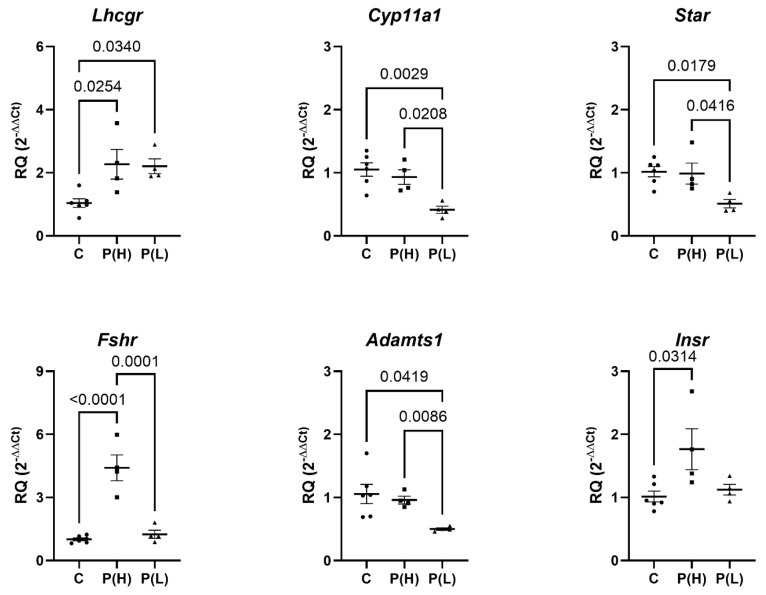
Expression of ovarian steroidogenesis and ovulation genes, as well as the insulin receptor gene, in the ovaries of female rats with DHEA-induced PCOS and high- or low-blood progesterone levels compared to age-matched controls. Expression of genes encoding the LHR (*Lhcgr*), FSH receptor (*Fshr*), cholesterol-transporting protein StAR (*Star*), cytochrome CYP11A1 (*Cyp11a1*), matrix metalloproteinase ADAMTS-1 (*Adamts1*), and insulin receptor (*Insr*) is shown. Data are presented as mean ± SEM. The number of animals in group C was *n* = 6, and in the P(H) and P(L) groups, *n* = 4 each.

An analysis of the expression of ovarian genes encoding key components of ovarian steroidogenesis and ovulation showed that, compared with the control, the expression of the gene encoding LHR was significantly increased in the ovaries of rats from the P(H) and P(L) groups, while the expression of the gene encoding the FSH receptor was increased only in the P(H) group (Figure 3).

In the P(L) group, the expression of the *Star*, *Cyp11*, and *Adamts1* genes was decreased when compared with both the control and the P(H) group (Figure 3), indicating a significant weakening of the expression of key genes of ovarian steroidogenesis and ovulation in rats from the PCOS(L) cohort. The expression of the insulin receptor gene slightly increased in the P(H) group (Figure 3), which is consistent with the data on the initial stages of IR development in this group (an increase in the insulin level and the IR index 120 min after a glucose load).

### 2.3. Evaluation of Ovarian Morphology in Female Rats with DHEA-Induced PCOS

To evaluate ovarian morphology, two groups from the high (P-C(H))- and low (P-C(L))-progesterone cohort were treated twice (24 h apart) with the gonadotropin-releasing hormone antagonist Cetrotide (50 μg/rat per injection, s.c.) to synchronize the estrous cycle and prevent the influence of endogenous gonadotropins on folliculogenesis and corpora lutea formation. Forty-eight hours after the first injection of Cetrotide, the ovaries of the animals were studied in comparison with control rats that received Cetrotide in the same doses (C-C) (Figure 1).

**Figure 4:**
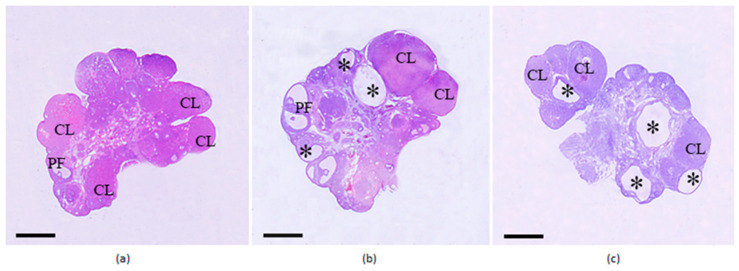
Ovarian sections from control female rats and from female rats with DHEA-induced PCOS and initially high- or low-progesterone levels. (**a**)—C-C group; (**b**)—P-C(H) group; (**c**)—P-C(L) group. Designations: PF—preovulatory follicles, CL—corpora lutea of the previous cycle, *—follicular cysts. Hematoxylin and eosin staining. Scale bar—1 mm.

In the ovaries of both groups of female rats with PCOS, large tertiary follicles were found, the number of which was significantly lower than in the ovaries of the control animals (Figure 4, Table 3). At the same time, the number of preovulatory follicles (PFs) did not differ between the control and PCOS groups. Infiltration of tertiary follicles and PF with leukocytes was shown, and macrophages were detected. In the control animals, an average of 8–9 layers of granulosa cells were detected in the PF wall; whereas in PCOS rats, the number of granulosa cell layers in the PF decreased to 2–3, indicating thinning of the follicular wall. The number of corpora lutea corresponding to the diestrus/proestrus phase (developed in the previous cycle) in the ovaries of rats belonging to groups C-C and P-C(H) did not differ significantly, but it was reduced in the P-C(L) group (Figure 4, Table 3). In control rats, the corpora lutea are dense, rounded, and located along the surface of the ovarian cortex, with degenerating luteal cells with large vacuoles present in their centers. In PCOS rats, the corpora lutea contain a significant number of degenerating luteal cells with large vacuoles, which are scattered throughout the structure. In isolated cases, very large corpora lutea are also observed, within which blood cells are visualized. Moreover, the corpora lutea in the ovaries of P-C(H) rats are significantly larger than those in the P-C(L) group and in control animals (Figure 4). In the control group, corpora lutea area ranged from 0.6 to 2.4 mm^2^ (mean 1.52 ± 0.22 mm^2^); in the P-C(L) group, corpora lutea area ranged from 0.9 to 3.1 mm^2^ (mean 2.21 ± 0.27 mm^2^); and in the P-C(H) group, corpora lutea area ranged from 2.7 to 4.5 mm^2^ (mean 3.56 ± 0.20 mm^2^) (*p* < 0.05 as compared to control and P-C(L) group). In both groups of PCOS rats, there are numerous follicular cysts, predominantly medium or large in size (Figure 4, Table 3).

The obtained data indicate the development of a multifollicular ovarian phenotype, and in rats with initially elevated progesterone levels, ovulatory activity is maintained, as indicated by a comparable number of corpora lutea to the control group, while in rats with initially reduced progesterone levels, ovulatory activity is significantly reduced.

### 2.4. Evaluation of the Effect of Ovulation Inducers—TP03 and hCG—On Progesterone and Estradiol Levels in PCOS Rats Pre-Treated with Follimag and Cetrotide

To evaluate the effect of ovulation inducers, PCOS rats were treated with the Follimag (20 IU/rat, s.c.), which was administered simultaneously with the first injection of Cetrotide (50 μg/rat per injection, s.c.), and 24 h later, Cetrotide was administered again. Forty-eight hours after Follimag treatment, the animals were treated with TP03 (25 mg/kg, i.p., in 200 μL of DMSO, 3 groups from each cohort), hCG (25 IU/rat, s.c., 3 groups from each cohort), or 200 μL of DMSO (one group from each cohort). The following groups of PCOS rats were formed (*n* = 4 in each): with DMSO injection—P-F(H) and P-F(L); with TP03 treatment and euthanasia after 4, 16, and 24 h—PT4(H), PT4(L), PT16(H), PT16(L), PT24(H), and PT24(L); and with hCG treatment and euthanasia after 4, 16 and 24 h—PG4(H), PG4(L), PG16(H), PG16(L), PG24(H), and PG24(L) (Figure 1).

**Figure 5:**
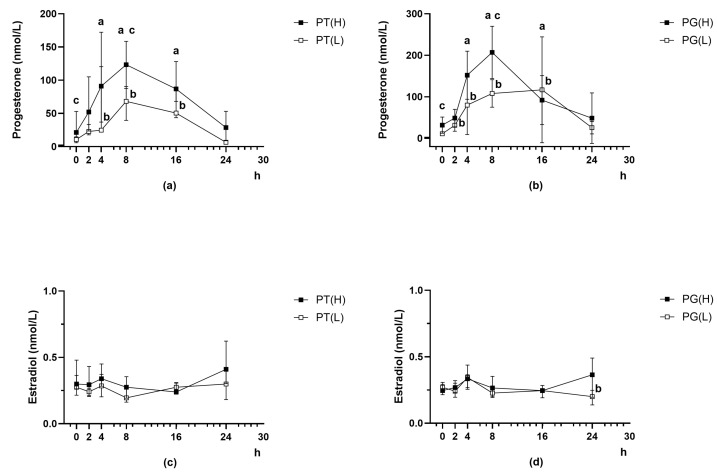
Dynamics of changes in progesterone (**a**,**b**) and estradiol (**c**,**d**) levels in the blood of PCOS rats with initially high or low progesterone levels when they were treated with LHR agonists: TP03 (**a**,**c**) or hCG (**b**,**d**). Data are presented as the median and interquartile range (25%; 75%). Differences with the P-F(H) group (^a^) for groups from the PCOS(H) cohort and with the P-F(L) group (^b^) for groups from the PCOS(L) cohort, as well as between groups from the PCOS(H) and PCOS(L) cohorts at the same time point (^c^), are significant at *p* < 0.05. In all groups: *n* = 4.

After treatment of PCOS rats with Follimag and Cetrotide (48 h after treatment with Follimag), progesterone levels were equalized in groups of animals from PCOS(H) and PCOS(L) cohorts (Figure 5; Table S1). Thus, analysis of progesterone levels in the formed groups of PCOS rats belonging to the same cohort revealed no significant differences between them (Table S1).

Ovulation inducers, TP03 (25 mg/kg, i.p.) or hCG (25 IU/rat, s.c.), increased blood progesterone levels in PCOS rats, both in groups with initially high and low hormone levels (Figure 5a,b). The stimulating effect of hCG and TP03 on progesterone production in rats from the PCOS(H) cohort was observed in the time interval from 4 to 16 h, while in rats from the PCOS(L) cohort, the stimulating effect of hCG developed earlier than the effect of TP03 (after 2 h), and both effects persisted for 16 h (Figure 5a,b). When making a pairwise comparison of the PT(H)/PG(H) or the PT(L)/PG(L) groups at one time point, it was shown that in the PG(H) group, 8 h after treatment with LHR agonists, the progesterone level was on average 61% higher than in the PT(H) group (*p* < 0.05), while in the PG(L) group, 8 and 24 h after such treatment, the level of this hormone was on average 67 and 95% higher than in the PT(L) group (*p* < 0.05). At the 8 h time point, both hCG and TP03 showed significant differences in their stimulating effects on progesterone levels between the PCOS(H) and PCOS(L) cohorts (Figure 5a,b). It should be noted that for hCG, the PCOS(H) and PCOS(L) cohorts showed differences in the dynamics of this effect: in the PCOS(H) cohort groups, hormone levels reached a maximum at 8 h and began to decrease by 16 h; in the PCOS(L) cohort groups, hormone levels at 8 and 16 h were similar (Figure 5b).

Blood estradiol levels in PCOS rats did not change significantly with either LHR agonist at all time points studied, with the exception of a decrease in blood hormone concentrations in PG24(L) rats compared to the baseline time point, and did not differ significantly between groups derived from the PCOS(H) and PCOS(L) cohorts (Figure 5c,d).

### 2.5. Evaluation of the Effect of TP03 and hCG on the Expression of Genes Involved in Steroidogenesis and Ovulation in the Ovaries of PCOS Rats

**Figure 6:**
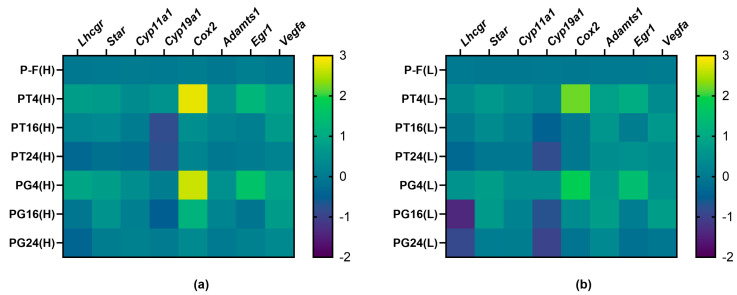
Heatmaps showing differential gene expression in the ovaries of PCOS rats and the effect of treatment with LHR agonists, TP03 and hCG, at different time intervals after their administration to animals from cohorts with high (**a**) and low (**b**) progesterone levels. The gene expression data are presented as log_10_-transformed Relative Quantification (RQ) values. Yellow, green, and blue shadings represent higher, average, and lower relative gene expression levels, respectively. In all groups: *n* = 4.

In the ovaries of PCOS rats, the expression of genes responsible for steroidogenesis and ovulation, as well as its changes at different time intervals after treatment with TP03 and hCG, were assessed using RT-PCR. It was shown that 4 h after treatment with TP03 and hCG, a significant increase in the expression of genes encoding LHR and key components of ovarian steroidogenesis, *Star* and *Cyp11a1*, was detected in the ovaries of PCOS rats belonging to the PCOS(H) cohort (Table 4, Figure 6). At 16 and 24 h after administration of both LHR agonists, the expression of the *LHR*, *Star*, and *Cyp11a1* genes did not differ from that in the P-F(H) group but decreased compared to the TP4(H) and TG4(H) groups, indicating a time-dependent attenuation of the stimulating effect of TP03 and hCG on ovarian steroidogenesis. At 16 and 24 h after treatment with TP03 (but not hCG), a significant decrease in aromatase gene expression was detected (Table 4, Figure 6). In the PCOS rat groups belonging to the PCOS(L) cohort, the same patterns of changes in the expression of ovarian steroidogenesis genes in response to stimulation with TP03 and hCG were observed as in the groups from the PCOS(H) cohort. At the same time, in this case, no significant increase in the expression of the *Lhcgr* gene was detected 4 h after treatment with LHR agonists (Table 4, Figure 6). It was also found that the expression of the *Lhcgr* gene in the PG16(L) and PG24(L) groups, 16 and 24 h after treatment with hCG, was lower than in the corresponding groups with TP03 treatment (Table 4, Figure 6). Thus, TP03 and hCG demonstrate the ability to stimulate the expression of genes involved in the synthesis of steroid hormones in PCOS rats with different initial progesterone levels, which is consistent with the data on the stimulation of progesterone levels in the blood of animals by these LHR agonists 4–16 h after such treatment.

Treatment with TP03 and hCG after 4 h resulted in an increase in the expression of the *Cox2*, *Adamts1*, *Egr1*, and *Vegfa* genes encoding cyclooxygenase-2, metalloproteinase ADAMTS-1, early growth response protein 1 (EGR-1), and vascular endothelial growth factor A (VEGF-A) in the ovaries of PCOS rats from the PCOS(H) cohort (Table 5, Figure 6). Moreover, the expression of the *Cox2* gene increased by an average of 440–650 times compared to the P-F(H) group, while the expression of the *Egr1* gene increased by 17–36 times. The expression of the *Cox2*, *Adamts1*, and *Egr1* genes decreased to the level in the P-F(H) group after 16 h, while the expression of the *Vegfa* gene remained elevated after 16 h (Table 5, Figure 6). In the PCOS rat groups from the PCOS(L) cohort, the pattern of ovarian gene expression regulation was qualitatively similar (Table 5, Figure 6).

### 2.6. Morphological Analysis of Ovaries in PCOS Rats and the Effect of Ovulation Inducers TP03 and hCG

**Figure 7:**
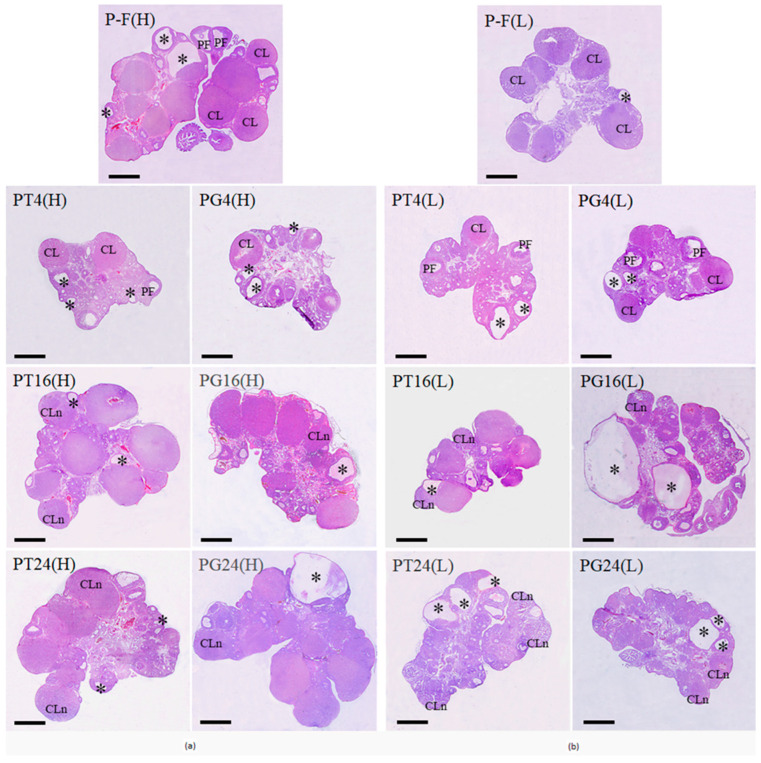
Ovarian sections from female rats with DHEA-induced PCOS with initially high (**a**) or low (**b**) progesterone levels treated with Follimag and Cetrotide, and the effects of LHR agonists, TP03, and hCG, at different time points (4, 16, and 24 h). Designations: PF—preovulatory follicle, CL—corpora lutea of the previous cycle, CLn—corpora lutea of the new generation, *—follicular cysts. Hematoxylin and eosin staining. Scale bar: 1 mm.

Morphological analysis of the ovaries in the P-F(H) and P-F(L) groups showed a tendency for an increased number of tertiary follicles (Figure 7), as compared to the corresponding groups treated with Cetrotide alone (Figure 4). When comparing the combined P-C(H)+P-C(L) groups and the P-F(H)+P-F(L) groups, the differences between them were significant (*p* = 0.014). At the same time, no differences were found in the number of PFs between these groups. The number of granulosa cell layers in the PF wall in the P-F(H) and P-F(L) groups, as well as in the P-C(H) and P-C(L) groups, was reduced (Figure 4 and Figure 7). However, in the ovaries of PCOS rats treated with Follimag, single PFs with normal follicular wall thickness, indistinguishable from the control, and intussusceptions were detected. Tertiary follicles and PFs in the P-F(H) and P-F(L) groups, like those in the P-C(H) and P-C(L) groups, were infiltrated with leukocytes and contained macrophages (Figure 7).

When rats with initially high progesterone levels were treated with TP03 and hCG, a significant decrease in the number of tertiary follicles was observed after 4 h and in the number of PFs after 24 h, indicating that most PFs had entered ovulation and was consistent with the appearance of new-generation corpora lutea after 16 h (Figure 7, Table 6). After 24 h, an average of 3–4 new-generation corpora lutea were detected in the ovaries of rats from the PT24(H) and PG24(H) groups, indicating successful ovulation (Figure 7, Table 6). Similar changes were shown for PCOS rats with initially low progesterone levels. In this case, a more pronounced decrease and even disappearance of PF in the rat ovaries was observed 16 and 24 h after treatment with LHR agonists, as well as the appearance of a comparable number of new-generation corpora lutea at the same time points (Figure 7, Table 6). Thus, the onset of ovulation in rats from the PCOS(L) cohort precedes that in rats from the PCOS(H) cohort. The number of follicular cysts in the ovaries of PCOS rats did not change significantly after treatment with either LHR agonist (Table 6).

## 3. Discussion

### 3.1. Differential Characterization of the DHEA-Induced PCOS Model Based on Differences in Blood Progesterone Levels

Since hyperandrogenism is the most common feature of PCOS in women [9,10,11], one approach to creating experimental animal models of this disease is their long-term treatment with androgens DHEA, testosterone, and 5α-dihydrotestosterone, as well as the aromatase inhibitor letrozole, which disrupts the conversion of androgens to estrogens [14,15]. DHEA, an adrenocortical androgen that is converted into androstenedione and testosterone by 3β- and 17β-hydroxysteroid dehydrogenases (3β-HSD and 17β-HSD), respectively, is most commonly used to induce PCOS [14]. DHEA-induced hyperandrogenism results in the development of polycystic ovary morphology, impaired folliculogenesis and ovulation, and moderate metabolic dysfunctions [32]. In this case, the age of the animals at which DHEA treatment begins, the duration of treatment, and the dosage of the drug are very important. In rats, DHEA treatment is usually started at early postnatal age (P22–P27), which facilitates monitoring of PCOS development and allows obtaining of sufficiently homogeneous groups of animals in terms of estrous cycle phase and hormonal status, but such “early” models differ significantly in etiology and pathogenesis from PCOS in humans [33,34,35,36,37]. Consequently, several research groups have proposed inducing PCOS with DHEA in prepubertal female rats (P42–P47) [16,17], a model that more closely recapitulates the development of the most common forms of PCOS in women. It should be noted, however, that in comparison with “early” DHEA models, “prepubertal” PCOS models are more heterogeneous and complex in terms of animal standardization and assessment of morphofunctional and hormonal changes in the ovaries during the development of PCOS, which was also observed in our work.

We found that the progesterone levels in the blood of prepubertal female rats treated with DHEA for 3 weeks varied significantly. Based on these data and taking into account our data on the comparative analysis of DHEA-induced “early” and prepubertal models of PCOS, where differentiation of prepubertal (but not immature) animals by progesterone levels was detected for the first time [38], all PCOS animals were divided into two cohorts: with high (PCOS(H)) and low (PCOS(L)) progesterone levels. The pattern of changes in a number of morphometric, biochemical, and morphological parameters in rats with PCOS depended on the level of progesterone (Table 7). According to our data, rats from the PCOS(H) cohort, unlike the PCOS(L) cohort, have higher estradiol levels and normal or increased (as compared to control) expression of ovarian genes involved in stimulating ovarian steroidogenesis (*Star*, *Cyp11a1*, *Fshr*) and ovulation (*Adamts1*). Thus, they are characterized by preserved functional activity of the steroidogenic system, which may be one of the reasons for the more intense formation of corpora lutea compared to the PCOS(L) cohort, where the expression of these ovarian genes is significantly reduced.

In humans, PCOS is a rather heterogeneous condition. In clinical practice, it is customary to distinguish four phenotypes of PCOS based on the combination of the classic Rotterdam criteria: phenotype A (classic): hyperandrogenism + ovulatory dysfunction + polycystic ovaries; phenotype B (anovulatory): hyperandrogenism + ovulatory dysfunction; phenotype C (ovulatory): hyperandrogenism + polycystic ovaries; phenotype D (non-androgenic): ovulatory dysfunction + polycystic ovaries [39]. However, it remains a subject of debate whether the formation of different phenotypes is based on distinct pathogenic mechanisms or whether, in the natural history of PCOS, different phenotypes succeed one another [40]. Our data indicate that treating rats with DHEA leads to the development of two distinct phenotypes, which may cautiously be termed “ovulatory” and “anovulatory”. This finding bears certain parallels to the formation of different PCOS phenotypes in humans.

Thus, rats with high progesterone levels had increases in both ovarian mass and its ratio to body mass, indicating ovarian hypertrophy and is considered a characteristic feature of PCOS. Moreover, the number of corpora lutea from previous cycles was comparable to that in controls, and their size significantly exceeded that of control animals and rats from the PCOS(L) cohort. This indicates relative preservation of ovulatory function in PCOS rats of the PCOS(H) cohort, despite cycle irregularity. It can be suggested that large corpora lutea are a potential source of excess progesterone production in the PCOS(H) cohort. Rats with low progesterone levels showed an increase in the specific mass of the ovaries while maintaining their total mass, as well as a decrease in the number of corpora lutea from previous cycles compared to controls, which brings this model closer to the anovulatory phenotype of PCOS in humans.

A tendency toward elevated blood insulin levels and a significant increase in the IR index 120 min after a glucose load in rats from the PCOS(H) cohort indicate decreased tissue sensitivity to insulin, which may predict metabolic disorders. Furthermore, Insr gene expression was increased in the ovaries of PCOS(H) rats, potentially representing a compensatory mechanism to maintain follicular cell insulin sensitivity during the early stages of systemic insulin resistance. In this regard, it is worth noting that *Insr* gene expression is also elevated in the ovaries of patients with PCOS, reflecting a pathogenetic link between ovarian insulin signaling and the development of this disease [41]. Similar information is lacking for experimental animals, but it has been shown that obesity, a pathogenetic factor in PCOS, also increases insulin receptor expression in the ovaries of mice, and this effect is tissue-specific [42].

There is evidence that long-term (35 days) DHEA treatment of female Sprague–Dawley rats leads to the development of obesity, IR, hyperinsulinemia, and increased oxidative stress [17]. However, it should be noted that, unlike the Wistar rats we used, the authors used Sprague–Dawley rats, which are predisposed to obesity, to induce PCOS, and they administered DHEA for a much longer period than in our case. The combination of DHEA treatment and a high-fat diet in rodents promotes the development of the pathogenetic picture of PCOS and accelerates the development of metabolic disorders [32]. One of the factors in this case is hyperinsulinemia, which, on the one hand, leads to the development of IR and, on the other hand, increases androgen production [15], thereby disrupting the maturation of antral follicles and causes their premature luteinization [43]. There is convincing clinical evidence of a close relationship between PCOS and metabolic disorders such as type 2 diabetes mellitus, metabolic syndrome [44,45], and non-alcoholic fatty liver disease [46], which are characterized by IR and hyperinsulinemia.

Along with their differences, PCOS rats from the PCOS(H) and PCOS(L) cohorts also had distinct similarities. They demonstrated decreased blood LH levels. Although this is not a diagnostic criterion for PCOS in clinical medicine, women with PCOS typically have elevated blood LH levels, and in girls, elevated LH levels can occur even in the prepubertal period, leading to hyperandrogenism and the development of the pathogenetic picture of PCOS [9,47,48]. Among the mechanisms of increasing LH levels, as well as the LH/FSH ratio in patients with PCOS, the key role is played by the following: (1) An increase in the frequency and amplitude of pulsatile GnRH releases by hypothalamic neurons, which leads to an abnormal increase in LH production by gonadotrophs of the anterior pituitary gland, but at the same time suppresses their release of FSH. (2) An increase in the sensitivity of LH-producing gonadotrophs to the stimulating effect of GnRH. (3) A weakening of the negative feedback system in the gonadal axis responsible for the inhibition of GnRH-expressing neurons by progesterone and estrogens [49,50]. Prenatal androgenization of rodents and monkeys also results in an increase in LH levels in adulthood with the development of the PCOS phenotype [51,52,53,54]. At the same time, in our study, PCOS was induced by a three-week administration of exogenous DHEA to prepubertal animals. We believe that DHEA-induced hyperandrogenism, which is confirmed by a significant increase in blood testosterone levels in PCOS rats, suppresses the synthesis and secretion of LH by gonadotrophs via a negative feedback mechanism. This resulted in a decrease in blood LH levels and an increase in ovarian LHR gene expression, which is compensatory in conditions of LH deficiency. In this regard, it is necessary to mention a decrease in the expression of FSH receptors in the P(H) group. This may be due to a decrease in FSH levels, which, however, was not assessed in this study. A decrease in LH levels in the blood of various strains of rats with PCOS, which were treated with DHEA in early postnatal (starting at the age of 22–25 days) and prepubertal age, was also observed by other authors [17,55].

### 3.2. Induction of Ovulation in Rats with DHEA-Induced PCOS Using Allosteric (TP03) and Orthosteric (hCG) LHR Agonists

One of the promising areas of research in the search for effective stimulators of ovarian steroidogenesis that can be used for controlled ovulation induction in ART for PCOS is the development of low-molecular-weight LHR agonists that target allosteric sites of the receptor [23,24,25]. Of greatest interest here are thieno[2,3-*d*]-pyrimidine derivatives, including the compound TP03 we developed. They penetrate through the external entrance into the internal cavity of the channel formed by the transmembrane domain of LHR and bind to an allosteric site located in the upper third of the channel. This activates the receptor, inducing its functional interaction with heterotrimeric G proteins, thus triggering LH-activated intracellular cascades involved in stimulating steroidogenic pathways in ovarian theca cells and testicular Leydig cells [24,25].

We have previously shown that TP03 and its structural homolog TP4/2 stimulate ovarian steroidogenesis and ovulation in healthy female rats [30,56], as well as in animals with type 2 diabetes mellitus [57]. In the present study, we show for the first time that TP03 triggers ovulation, increases progesterone production, and induces corpora lutea formation in prepubertal female rats with DHEA-induced PCOS, with comparable efficacy in cohorts with both high and low progesterone levels. To achieve this, as shown in Figure 1 and described in detail in the Materials and Methods section (Section 4.4), we randomly formed the required number of groups (each with four animals) from cohorts with high and low progesterone levels, which then received treatment with LHR agonists or placebo.

Progesterone levels increased 8 h after the start of TP03 treatment of PCOS rats, which is consistent with an increase in the expression of the gene encoding LHR, responsible for the recognition of the hormonal stimulus (gonadotropin or low-molecular-weight LHR agonist), and the genes encoding StAR protein and cytochrome CYP11A1, which catalyze the initial, rate-limiting stages of progesterone synthesis, in the ovaries 4 h after such treatment. At 16 and 24 h after TP03 treatment, aromatase gene expression also decreased, which is associated with the switch of steroidogenic pathways to progesterone synthesis during ovulation. For groups from the high-progesterone cohort, the decrease in *Cyp19a1* expression was significant compared to the P-F(H) group. Furthermore, 4 h after treatment with TP03, the expression of the *Cox2*, *Egr1*, *Adamts1*, and *Vegfa* genes increased in the ovaries of PCOS rats. These genes are involved in preparing the oocyte for ovulation and ensuring subsequent follicular rupture. This preceded the formation of new-generation corpora lutea and ovulation. Increased expression of several genes (*LHR*, *Cox2*, *Egr1*) was more pronounced in PCOS rats belonging to the PCOS(H) cohort, and this was associated with a more pronounced increase in progesterone levels upon stimulation with TP03. Furthermore, the number of corpora lutea formed in the ovaries of PCOS rats upon stimulation with TP03 and hCG was comparable. Only some differences in the dynamics of their formation were revealed, since in the groups from the PCOS(L) cohort, the number of corpora lutea after 16 and 24 h was comparable, while in the groups from the PCOS(H) cohort, they were detected mainly after 24 h. This did not depend on the type of LHR agonist, but it was determined by belonging to a particular cohort. The totality of the obtained data indicates the absence of fundamental differences in the response of the ovaries of PCOS rats to hCG and TP03, confirming the comparable effectiveness of using TP03 as an ovulation trigger compared to hCG in PCOS.

Prior to our studies, there was no information on ovarian stimulation with low-molecular-weight allosteric LHR agonists under conditions of reproductive dysfunction, including PCOS. Despite the comparable efficacy demonstrated here, TP03 offers several potential advantages over gonadotropins that warrant further investigation in PCOS models. First, unlike hCG, allosteric agonists do not cause hyperactivation of the LHR, as we previously demonstrated for TP03 and its structural homolog TP4/2 in activating steroidogenesis in the ovaries and testes [30], and as others have demonstrated for Org43553, another thieno[2,3-d]-pyrimidine derivative [29]. This may prevent the risk of OHSS when using allosteric agonists for controlled ovulation induction. Secondly, repeated administration of TP03 or TP4/2 over several days does not lead to desensitization of LHR or a decrease in its expression in target tissues [27,28], which may be due to their moderate stimulatory effect on receptor activity and, presumably, a shift in intracellular signaling aimed mainly at activating Gs proteins and cAMP-dependent pathways [24,25,26]. Thirdly, low-molecular-weight LHR agonists have the properties of LHR-selective low-molecular-weight chaperones and are therefore capable of activating mutant receptors with a reduced ability to translocate to the plasma membrane, as shown for Org42599 (Org 43553 trifluoroacetate) [58]. This requires further research and may significantly expand the indications for their use in forms of PCOS associated with mutations in LHR [59,60]. Furthermore, TP03 and its structural homologs (TP4/2, Org43553) retain activity when administered orally, as they are stable and well absorbed from the gastrointestinal tract [29,30,31,61], significantly expanding the potential for their clinical application.

It should also be noted that there was no information on stimulation of steroidogenesis and ovulation by gonadotropin preparations in rodents with DHEA-induced PCOS model, with the exception of indirect data on ovulation induction in mice with DHEA-induced PCOS using GnRH [62]. For other PCOS models, information on ovarian stimulation by gonadotropins is also very limited and contradictory. Thus, a stimulating effect of equine chorionic gonadotropin on the process of folliculogenesis was shown in rats with PCOS induced by 5α-dihydrotestosterone [63,64]. But in one study, this was accompanied by the appearance of new-generation corpora lutea and a decrease in the number of follicular cysts [63], and in another study, corpora lutea were not detected [64], despite the same rat strain and age, equal equine chorionic gonadotropin dosage, and the 5α-dihydrotestosterone treatment approach. The discrepancies found between studies may be due to differences in the timing of tissue sampling (0–30 h in [63] versus 48 h in [64]) and the duration of androgen administration (12 weeks in [63] versus 4 weeks in [64]), which significantly influences the severity of PCOS and the ovarian response to gonadotropin stimulation.

## 4. Materials and Methods

### 4.1. Experimental Animals

Prepubertal female Wistar rats were used, which were 42–45 days old at the beginning of the experiment. The animals were obtained from the Rappolovo nursery (Leningrad Region, St. Petersnurg, Russia). They were kept in individual cages, 5–6 animals per cage, with free access to standard food and water, under a 12 h day/12 h night cycle and a temperature of 22–24 °C. The animal experiments were approved by the Bioethics Committee of the Institute of Evolutionary Physiology and Biochemistry of the Russian Academy of Sciences (Protocol #1-4/2025 of the Bioethics Committee session-1 (30 January 2025) of IEPhB RAS), The Guide for the Care and Use of Laboratory Animals, and Directive 2010/63/EU of The European Parliament and of The Council of 22 September 2010 on the protection of animals used for scientific purposes. All the efforts were made to minimize animal suffering and reduce the number of experimental animals. For local anesthesia during tail vein blood collection, a 2% lidocaine solution (2–4 mg/kg) was applied. At the end of the experiment, the rats were anesthetized with isoflurane (“Karizoo”, Barcelona, Spain; 5% air mixture for induction of anesthesia and 2% air mixture for maintenance inhalation until euthanasia) using the Vet Optima small anesthesia system (“Zoomed”, St. Petersburg, Russia), and then the anesthetized animals were euthanized by decapitation.

### 4.2. Reagents and Drugs

The compound TP03, 5-amino-*N-tert*-butyl-2-(methylsulfanyl)-4-(3-(nicotinamido)phenyl)thieno[2,3-d]-pyrimidine-6-carboxamide, with LHR allosteric agonist activity, was synthesized using a modification of the Hanssen and Timmers method [65], as described previously [28]. TP03 was characterized by reversed-phase high-performance liquid chromatography (HPLC) and high-resolution mass spectrometry. According to HPLC analysis, the purity of TP03 was at least 99%. According to high-resolution mass spectrometry data, the molecular ion mass [M + Na^+^] for TP03 was 515.1304 (calculated value: 515.1294). The high-resolution mass spectra (ElectroSpray Ionization-Time Of Flight, ESI-TOF) were recorded using a “Bruker micrOTOF” spectrometer (“Bruker”, Karlsruhe, Germany).

Follimag, a gonadotropin isolated from pregnant mares’ serum with a specific activity of 1000 IU/vial and follicle-stimulating hormone (FSH)-like activity, was purchased from “Mosagrogen” (Moscow, Russia), and hCG was obtained from “Moscow Endocrine Factory” (Moscow, Russia). Cetrotide (cetrorelix), a gonadotropin-releasing hormone antagonist (purity of ≥98%), was purchased from “Macklin Biochemical Technology Co. Ltd.” (Shanghai, China), and DHEA (purity of ≥99%) was purchased from “ACMEC Biochemical Co. Ltd.” (Shanghai, China). Other reagents were purchased from “Sigma-Aldrich” (St. Louis, MO, USA), “Vecton” (St. Petersburg, Russia), and “NevaReaktiv” (St. Petersburg, Russia).

### 4.3. Evaluation of Hormone Concentrations and Intraperitoneal Glucose Tolerance Test (iGTT)

The concentration of ovarian steroid hormones—progesterone, estradiol, and testosterone—was assessed using the Progesterone-ELISA and Estradiol-ELISA kits from “XEMA” (Moscow, Russia) and the Steroid ELISA-Testosterone kit from “Alkor-Bio Company Ltd.” (Moscow, Russia). Blood insulin, leptin, and LH concentrations were measured using the “ELISA kit for Insulin, Rat”, “ELISA kit for Leptin, Rat”, and “ELISA kit for LH, Rat” (Cloud-Clone Corp., Katy, TX, USA), respectively. Blood glucose concentrations were assessed using a glucometer (LifeScan Johnson & Johnson, Milpitas, CA, USA) and One Touch Ultra test strips (LifeScan Inc., Malvern, PA, USA).

The iGTT was performed by intraperitoneally administering a 40% glucose solution (2 g/kg) to rats. Glucose levels were monitored before administration (baseline) and 15, 30, 60, 90, and 120 min after glucose administration. Insulin and leptin levels were assessed before (baseline) and 120 min after the glucose load. Before the iGTT, rats were fasted for 10 h, with free access to water. For the glucose concentration (mmol/L) vs. time (min) curves obtained during the iGTT, the AUC_0–120_ value, representing the integrated area under these curves, was calculated and expressed in arbitrary units. The insulin resistance (IR) index was calculated as the product of the concentrations of glucose and insulin in the blood before (IR_0_ index) and 120 min after the glucose load (IR_120_ index), and it was expressed in arbitrary units.

### 4.4. Induction of PCOS in Prepubertal Female Rats and Their Treatment with Gonadotropins and TP03

To induce PCOS, female Wistar rats (*n* = 86, 42–45 days old) were treated with DHEA (6 mg/100 g body weight/day, s.c.) as a solution in sesame oil (200 μL) for 3 weeks. Control female Wistar rats of the same age (*n* = 11) received sesame oil (s.c.) instead of DHEA in the same volume. During the last 10 days of DHEA treatment, vaginal smears were taken from female rats to assess the phases of the estrous cycle. Rats with an irregular estrous cycle were considered rats with developed PCOS, which meets the criteria presented in the study [17,66]. In total, 72 female rats (84% of the initial number of animals taken) had an irregular estrous cycle. At the end of the third week, blood progesterone and estradiol levels were assessed. Evaluation of progesterone concentrations in PCOS rats revealed significant variability, with animals forming two cohorts—with high and low hormone levels (with a median of 56.3 nmol/L). This is consistent with our earlier data obtained in a comparative analysis of DHEA-induced “early” and prepubertal models of PCOS [38] and shows that prepubertal PCOS rats with low and high progesterone levels differ significantly in several parameters important for assessing ovarian steroidogenesis and ovulation induction. These parameters may correspond to different phenotypes of DHEA-induced PCOS in rats, which served as the basis for their separate analysis in the present study.

Accordingly, PCOS rats were divided into two equal cohorts—with progesterone levels above (PCOS(H)) and below (PCOS(L)) the median value (36 animals in each). Each cohort was then randomly divided into 9 groups (4 rats in each). There were no differences in progesterone levels between the groups in each cohort. Two groups, including PCOS rats from the cohorts with high (group P(H), *n* = 4) and low (group P(L), *n* = 4) progesterone levels, were used to characterize the PCOS model by morphometric, metabolic, and hormonal parameters. They were compared with control female rats treated with sesame oil, which were in the diestrus phase of the estrous cycle (group C, *n* = 6) (Figure 1). Two more groups from the cohorts with high (group P-C(H), *n* = 4) and low (group P-C(L), *n* = 4) progesterone levels were treated with Cetrotide (cetrorelix), a gonadotropin-releasing hormone antagonist (50 μg/rat per injection, s.c.), at a 24 h interval to synchronize the estrous cycle and prevent the production of endogenous gonadotropins. Forty-eight hours after the first Cetrotide treatment, they were anesthetized and ovarian morphology was assessed, compared with control rats treated with Cetrotide according to the same scheme (group C-C, *n* = 5) (Figure 1). The remaining PCOS rats were treated with the folliculogenesis activator Follimag (20 IU/rat, s.c.), which was administered simultaneously with the first injection of Cetrotide (50 μg/rat per injection, s.c.), and 24 h later, Cetrotide was administered again at the same dose. Forty-eight hours after Follimag treatment, the PCOS rats were given a single injection of TP03 (25 mg/kg, i.p., in 200 μL of DMSO, 3 groups from each cohort) or hCG (25 IU/rat, s.c., 3 groups from each cohort) or 200 μL of DMSO, TP03 solvent (one group from each cohort) (Figure 1). We previously demonstrated that DMSO at the specified volume (200 µL) has no significant effect on steroid hormone levels, the expression of genes involved in steroidogenesis, and ovarian morphology in female rats [30], nor on metabolic and hormonal parameters and testicular morphology in male rats [27,28]. This minimizes the potential risks of DMSO administration on the parameters assessed in PCOS rats.

PCOS rats treated with TP03 or hCG were euthanized 4, 16, and 24 h after administration of LHR agonists to assess the dynamics of changes in hormonal status, ovarian gene expression, and the formation of preovulatory follicles (PFs) and corpora lutea in the ovaries. Rats treated with DMSO were euthanized immediately after solvent administration. Thus, depending on the type of treatment, time point and cohort, PCOS(H), or (PCOS(L), the following groups of PCOS animals were formed (*n* = 4 in all groups): with DMSO injection—groups P-F(H) and P-F(L); with TP03 treatment and euthanasia of animals after 4, 16, and 24 h—groups PT4(H), PT4(L), PT16(H), PT16(L), PT24(H), and PT24(L); and with hCG treatment and euthanasia of animals after 4, 16, and 24 h—groups PG4(H), PG4(L), PG16(H), PG16(L), PG24(H), and PG24(L) (Figure 1). Blood samples for progesterone and estradiol levels were collected in the following order: at baseline for the PT4(H), PT16(H), and PT24(H) groups (*n* = 12), after 2 h for the PT4(H) and PT16(H) groups (*n* = 8), after 4 h for the PT4(H) and PT24(H) groups (*n* = 8), after 8 h for the PT16(H) and PT24(H) groups (*n* = 8), after 16 h for the PT16(H) group (*n* = 4), and after 24 h for the PT24(H) group (*n* = 4). A similar blood sampling scheme was used for the other cohort and in the case of hCG treatment. This blood sampling scheme minimized animal stress, as no more than four blood samples were collected from any individual rat. Blood was collected from the tail vein under local anesthesia, as described in Section 4.1.

### 4.5. Determining the Phase of the Estrous Cycle

The estrous cycle phases in female rats were assessed by microscopic examination of vaginal smears, according to standard methods [67,68]. Smears were collected daily at 11:00 AM, with the anterior body restrained and the vaginal vestibule inspected. To obtain a smear, a cotton swab soaked in saline was inserted into the vagina, rotated several times, and the smear was transferred from the swab to a glass slide and air-dried. Estrous cycle phases were determined based on the ratio of nucleated epithelial cells, anucleated keratinized epithelial cells, and leukocytes.

### 4.6. Evaluation of Ovarian Gene Expression

Quantitative RT-PCR was used to study ovarian gene expression. Total RNA was isolated from the ovaries using the ExtractRNA reagent (TRIzol analog) (“Evrogen”, Moscow, Russia), and reverse transcription was performed using the MMLV RT Kit (“Evrogen”, Moscow, Russia). RT-PCR was performed using an Applied Biosystems^®^ 7500 Real-Time PCR System (“Life Technologies, Thermo Fisher Scientific Inc.”, Waltham, MA, USA) in a mixture containing 0.04 μM forward and reverse primers, as well as using the qPCRmix-HS SYBR+Low ROX kit (“Evrogen”, Moscow, Russia). To assess the stability of reference genes, raw C_t_ (cycle threshold) data were imported into the RefFinder online tool (https://www.ciidirsinaloa.com.mx/RefFinder-master (accessed on 10 June 2025). RefFinder utilizes four commonly used algorithms for reference gene validation (geNorm, NormFinder, BestKeeper, and the comparative Delta-C_t_ method). The detailed validation analysis is presented in the Supplementary Materials (Figure S1). Based on this analysis, the *Actb* and *18S rRNA* genes were selected as reference genes from four candidate genes (*Actb*, *18S rRNA* (*rn18S*), *Hprt*, and *Gapdh*) and then used to study ovarian gene expression. The list of primers used, including those for the reference genes, is given in the Supplementary Materials (Table S2). The delta-delta C_t_ method was used for the calculation [69], presenting the data as a multiple of the gene expression value in the experimental group relative to the value of its expression in the control, using 7500 Software v2.0.6 and Expression Suite Software v1.0.3 (USA).

### 4.7. Morphological Analysis of the Ovaries and Identification of Follicular Cysts and Corpora Lutea

Morphological analysis of the ovaries was performed as described previously [30] in the C-C, P-C(H), and P-C(L) groups, as well as in the PCOS rats treated with ovulation inducers (TP03, hCG) compared to the P-F(H) and P-F(L) groups. For this purpose, the ovaries were fixed (48 h, 4 °C) in a 4% solution of *para*-formaldehyde (“Sigma”, St. Louis, MO, USA) in Na-phosphate buffer (20 mM, pH 7.4, 0.9% NaCl). After washing with the same buffer, they were placed in a 30% sucrose solution (4 °C) and then frozen on dry ice using Tissue-Tek^®^ medium (“Sacura, Finetek Europe”, Alphen aan den Rijn, The Netherlands). Longitudinal sections (10 μm thick) were obtained using a Leica CM-1520 cryostat (“Leica Microsystems”, Wetzlar, Germany). Every fourth or fifth section in the series was mounted on a Super-Frost slide (“Menzel”, Berlin, Germany). After washing with phosphate buffer and treating with 50% ethanol for 3 min, the sections were stained with hematoxylin and eosin and then mounted under a coverslip using glycerol. Sections were analyzed using a Carl Zeiss Imager A1 microscope (“Carl Zeiss”, Oberkochen, Germany) and Zen 3.4 software. The number of tertiary follicles, PFs, corpora lutea, and follicular cysts was counted in each section. These sections were analyzed by two independent observers. Only PFs in which an oocyte was visible were counted. The assessed parameters were presented as the number of structures (tertiary follicles, PFs, corpora lutea, and follicular cysts) per ovary. To estimate the size of the corpora lutea from the previous cycle, two of their diameters (the smaller and the larger) were measured at the section where they were at their maximum size using Zen 3.4 Blue Edition software. The area of the corpora lutea was determined using the formula for the area of an ellipsoid (S = π × D1 × D2/4), where D1 and D2 are the larger and smaller diameters of the ellipsoid. To obtain illustrations, slides with sections were scanned using a CanoScan 8800F scanner (“Canon”, Tokyo, Japan) and processed using Photoshop CS6.

### 4.8. Statistical Analysis of Results

Statistical analysis of the data was performed using IBM SPSS Statistics 26 (“IBM”, Armonk, NY, USA) and GraphPad Prism 10.5.0 (“GraphPad Software, Inc.”, La Jolla, CA, USA). Normality of distribution was tested using the Shapiro–Wilk test, while equality of variances was tested using the Levene test. In case of comparison of three or more groups under the condition of normal distribution, one-way analysis of variance (ANOVA) was used with post hoc tests—Bonferroni for data with equal variances, Tamhane’s test for data with unequal variances—presenting as mean ± SEM. Pairwise comparison was performed using the *t*-test for two independent samples. In case of non-normal distribution, the Kruskal–Wallis test was used for several independent samples, followed by pairwise comparison using the Mann–Whitney U-test with Bonferroni correction to adjust for multiple comparisons. Non-parametric test data were presented as median and interquartile ranges (25%; 75%). Differences were considered as significant at a significance level of *p* < 0.05.

## 5. Conclusions

Thus, we have shown for the first time that the thieno[2,3-d]-pyrimidine derivative TP03, endowed with LHR agonist activity, exerts a pronounced stimulating effect on ovarian steroidogenesis and ovulation in female rats with various phenotypes of DHEA-induced PCOS; thus, it is not inferior to hCG, the ‘gold standard’ of ovulation inducers. It increases progesterone levels, enhances the expression of ovarian steroidogenesis and ovulation genes, and induces the formation of corpora lutea in the ovaries of animals belonging to two different cohorts—with initially low and high progesterone levels—which have a different spectrum of ovarian functional disorders corresponding to moderate (high-progesterone model) and more pronounced (low-progesterone model) signs of PCOS. In this regard, it is necessary to emphasize that there are clinical studies demonstrating a relationship between progesterone levels and the success of ART procedures, not at the stage of ovulation induction but at the stage of embryo implantation and the development of clinical pregnancy [70,71].

The obtained results on the ability of TP03 to stimulate ovarian steroidogenesis and ovulation in rats with DHEA-induced PCOS indicate its potential for use in PCOS, including controlled ovulation induction in assisted reproductive technologies. However, before proceeding to clinical trials, confirmation of TP03’s safety for the fetus and a study of pregnancy outcomes following TP03-induced ovulation in experimental models are required, as well as a comparison of the effects of TP03, hCG, and GnRH agonists on the incidence of ovarian hyperstimulation syndrome. Long-term administration of effective doses of TP03 and its analogs to male rats for 5 days or more revealed no desensitization of LH receptors, no weakening of LH-dependent signaling, no structural changes in the testes, and no negative effects on spermatogenesis, indicating low risks of long-term use [27,28]. Further studies in females, including those utilizing oral administration, are warranted, as TP03 is resistant to degradation and is well-absorbed from the gastrointestinal tract, a feature that would significantly broaden its clinical applicability.

## 6. Limitations

Dividing PCOS rats into two cohorts, with high and low progesterone, does not meet the accepted criteria for different PCOS phenotypes in humans, as blood progesterone levels are not included in the classic criteria for this disease. However, our experimental data indicate a possible important role for progesterone levels in the etiology and pathogenesis of different PCOS phenotypes, which may be important for the success of in ART procedures in PCOS.

In the DHEA-induced rat model of PCOS we studied, blood LH levels and the expression of several ovarian steroidogenesis genes (in the PCOS(L) cohort) are reduced. This may be due to the triggering of negative feedback mechanisms in the gonadal axis after prolonged use of relatively high doses of exogenous DHEA. This differentiates this model, like other androgen-induced models of PCOS, from the disease in humans. This should be taken into account when comparing DHEA-induced PCOS in rats and the corresponding disease in humans.

## Figures and Tables

**Table 1:** Body weight and levels of progesterone and estradiol in control female rats and animals with DHEA-induced PCOS.

Indicator	C, *n* = 11	PCOS, *n* = 72	PCOS(H), *n* = 36	PCOS(L), *n* = 36
Body weight, g	196.6 ± 4.9	191.9 ± 2.3	198.2 ± 3.0	185.4 ± 3.1 #
Progesterone, nmol/L	16.5 (12.3; 34.1)	56.3 (18.7; 170.9)	170.7 (96.4; 212.3) *	18.8 (9.5; 29.3) #
Estradiol, nmol/L	0.29 (0.24; 0.35)	0.35 (0.26; 0.55)	0.53 (0.28; 0.77) *	0.30 (0.25; 0.38) #

Note. Differences with the control (*) and between the P(H) and P(L) groups (#) are significant at *p* < 0.05. Body weight values are presented as mean ± SEM (normally distributed), and the progesterone and estradiol levels are presented as the median and interquartile range (25%; 75%) (non-normally distributed).

**Table 2:** Body and ovarian weight, blood steroid hormone and gonadotropin levels, glucose homeostasis parameters, insulin and leptin levels, and insulin resistance index in groups of female rats with DHEA-induced PCOS with different progesterone levels compared to age-matched control animals.

Indicator	C, *n* = 6	P(H), *n* = 4	P(L), *n* = 4
Body weight, g	201.5 ± 8.1	192.0 ± 4.2	193.5 ± 9.4
Ovarian weight, mg	53.8 ± 4.1	76.7 ± 2.6 *	67.6 ± 3.8
Ovarian weight/body weight, arb. units	2.66 ± 0.13	4.00 ± 0.12 *	3.49 ± 0.09 *
Progesterone, nmol/L	20.6 (13.2; 41.9)	228.2 (87.5; 317.6) *	28.4 (27.2; 34.9) #
Estradiol, nmol/L	0.31 ± 0.04	0.65 ± 0.12 *	0.35 ± 0.06 #
Testosterone, nmol/L	18.6 ± 1.2	40.3 ± 1.4 *	31.7 ± 4.4 *
LH, ng/mL	19.3 ± 1.1	12.6 ± 0.9 *	12.3 ± 1.4 *
Glucose, mM (0)	4.3 ± 0.2	4.7 ± 0.4	4.8 ± 0.3
Glucose, mM (120)	5.2 ± 0.3	5.9 ± 0.1	5.4 ± 0.3
AUC_0–120_ (glucose), arb. units	1207 ± 45	1317 ± 116	1275 ± 90
Insulin, ng/mL (0)	0.63 ± 0.12	0.91 ± 0.12	0.60 ± 0.14
Insulin, ng/mL (120)	0.75 ± 0.11	1.39 ± 0.17	1.02 ± 0.24
IR_0_ index, arb. units	2.69 ± 0.48	4.23 ± 0.53	3.02 ± 0.90
IR_120_ index, arb. units	3.80 ± 0.46	8.15 ± 1.08 *	5.40 ± 1.06
Leptin, ng/mL (0)	1.79 ± 0.34	2.36 ± 0.36	1.83 ± 0.38
Leptin, ng/mL (120)	2.70 ± 0.54	4.21 ± 0.84	2.74 ± 0.38

Note. Differences compared to control (*) and between P(H) and P(L) groups (#) are statistically significant at *p* < 0.05. All data were normally distributed and are presented as mean ± SEM (normally distributed), except for progesterone levels, which were not normally distributed and were presented as the median and interquartile range (25%; 75%).

**Table 3:** Number of tertiary and preovulatory follicles, corpora lutea of the previous cycle, and follicular cysts in the ovaries of control rats and animals with PCOS, with initially different levels of progesterone.

Indicator	C-C, *n* = 5	P-C(H), *n* = 4	P-C(L), *n* = 4
Tertiary follicles, numbers	23.0 (14.0; 25.0)	8.5 (6.3; 10.0) *	5.0 (3.5; 8.8) *
Preovulatory follicles, numbers	2.80 ± 0.97	4.00 ± 1.41	1.50 ± 0.50
Corpora lutea of the previous cycle, numbers	5.60 ± 0.51	4.50 ± 0.50	2.75 ± 0.48 *
Follicular cysts, numbers	0.00 ± 0.00	5.00 (3.00; 7.75) *	5.00 (3.50; 6.50) *

Note. The number of PFs and corpora lutea of the previous cycle is presented as mean ± SEM (normally distributed), and the number of tertiary follicles and follicular cysts are presented as the median and interquartile range (25%; 75%) (non-normally distributed). *—the difference with group C-C is significant at *p* < 0.05.

**Table 4:** Expression of genes encoding the LH receptor and key enzymes of steroidogenesis in the ovaries of PCOS rats and the effect of treatment with LHR agonists, TP03 and hCG, at different time intervals after their administration to animals.

Group	*Lhcgr*	*Star*	*Cyp11a1*	*Cyp19a1*
High-progesterone cohort, PCOS(H)				
P-F(H)	0.99 ± 0.09	1.04 ± 0.17	1.05 ± 0.18	1.04 ± 0.16
PT4(H)	5.59 ± 0.86 ^a^	4.69 ± 1.00 ^a^	2.32 ± 0.22 ^a^	3.38 ± 1.03
PT16(H)	1.82 ± 0.83 ^b^	2.06 ± 0.29 ^b^	1.14 ± 0.28 ^b^	0.15 ± 0.03 ^a^
PT24(H)	0.46 ± 0.15 ^c^	0.71 ± 0.07 ^c^	0.56 ± 0.08 ^c^	0.17 ± 0.10 ^a^
PG4(H)	7.96 ± 1.72 ^a^	5.57 ± 1.17 ^a^	2.54 ± 0.48 ^a^	1.25 ± 0.09
PG16(H)	0.85 ± 0.25 ^b^	3.12 ± 0.65	1.29 ± 0.34	0.29 ± 0.10
PG24(H)	0.41 ± 0.15 ^c^	1.21 ± 0.07 ^c,d^	1.41 ± 0.35	1.09 ± 0.58
Low-progesterone cohort, PCOS(L)				
P-F(L)	1.04 (0.90; 1.18)	0.99 ± 0.03	0.97 ± 0.04	1.01 (0.85; 1.20)
PT4(L)	2.44 (2.28; 4.63)	4.34 ± 1.37 ^a^	2.48 ± 0.65 ^a^	1.68 (1.24; 5.38)
PT16(L)	1.05 (0.61; 1.20)	2.32 ± 0.37	1.31 ± 0.33	0.35 (0.09; 2.11)
PT24(L)	0.47 (0.34; 0.58) ^c^	0.88 ± 0.31	0.91 ± 0.19	0.15 (0.06; 0.38)
PG4(L)	3.49 (1.98; 10.12)	5.53 ± 2.12 ^a^	2.53 ± 0.71 ^a^	2.55 (1.93; 9.01)
PG16(L)	0.04 (0.03; 0.07) ^b,d^	4.65 ± 0.82 ^d^	1.45 ± 0.17	0.18 (0.03; 0.62)
PG24(L)	0.13 (0.07; 0.17) ^d^	1.17 ± 0.06	1.25 ± 0.09	0.10 (0.06; 0.12) ^c^

Note. Differences with the P-F(H) or P-F(L) group (^a^), between the PT4(H) and PT16(H) groups or between the PG4(H)/PG4(L) and PG16(H)/PG16(L) groups (^b^), between the PT4(H)/PT4(L) and PT24(H)/PT24(L) groups or between the PG4(H)/PG4(L) and PG24(H)/PG24(L) groups (^c^), as well as between the groups treated with TP03 or hCG at the same time point (PT24(H) vs. PG24(H), PT16(L) vs. PG16(L), and PT24(L) vs. PG24(L)) (^d^) are significant at *p* < 0.05. The gene expression, quantified in Relative Quantification (RQ) values, is presented as mean ± SEM (normally distributed), except for the expression of *Lhcgr* and *Cyp19a1* genes in the PCOS(L) cohort, where its values are presented as the median and interquartile range (25%; 75%) (non-normally distributed). In all groups: *n* = 4.

**Table 5:** Expression of genes encoding markers of ovulation and angiogenesis in the ovaries of PCOS rats and the effect of treatment with LHR agonists, TP03 and hCG, at different time intervals after their administration to animals.

Group	*Cox2*	*Adamts1*	*Egr1*	*Vegfa*
High-progesterone cohort, PCOS(H)				
P-F(H)	1.22 ± 0.33	0.98 ± 0.15	1.29 (0.38; 2.62)	1.03 ± 0.12
PT4(H)	647.7 ± 111.6 ^a^	3.20 ± 0.59 ^a^	18.5 (8.44; 26.5) ^a^	6.94 ± 0.96 ^a^
PT16(H)	2.69 ± 1.03 ^b^	1.67 ± 0.37 ^b^	1.29 (0.62; 1.82)	4.72 ± 0.81 ^a^
PT24(H)	1.65 ± 0.55 ^d^	0.96 ± 0.05 ^d^	1.13 (0.48; 2.39)	1.59 ± 0.26 ^c,d^
PG4(H)	445.7 ± 142.9 ^a^	2.99 ± 0.60 ^a^	35.8 (19.8; 48.8) ^a^	7.32 ± 1.63 ^a^
PG16(H)	15.35 ± 7.54 ^b^	1.67 ± 0.30	0.81 (0.74; 2.99) ^b^	5.31 ± 0.56 ^a^
PG24(H)	2.17 ± 0.82 ^d^	1.04 ± 0.15 ^d^	1.42 (0.62; 1.50) ^d^	2.20 ± 0.32 ^d^
Low-progesterone cohort, PCOS(L)				
P-F(L)	0.99 ± 0.12	1.00 (0.91; 1.09)	1.02 ± 0.18	1.12 (0.84; 1.47)
PT4(L)	161.3 ± 58.5 ^a^	5.94 (1.76; 12.17) ^a^	10.91 ± 1.45 ^a^	2.40 (2.02; 6.17)
PT16(L)	0.95 ± 0.42 ^b^	3.77 (3.69; 5.26) ^a^	1.25 ± 0.37 ^b^	4.33 (1.71; 5.22)
PT24(L)	0.97 ± 0.09 ^d^	2.51 (2.36; 4.64)	2.95 ± 1.26 ^d^	2.27 (1.29; 2.42)
PG4(L)	67.64 ± 30.42 ^a^	4.33 (1.34; 12.04)	28.13 ± 10.49 ^a^	2.99 (2.03; 11.00)
PG16(L)	2.41 ± 0.62	5.72 (4.11; 7.93) ^a^	1.18 ± 0.31 ^b^	5.46 (4.82; 8.25) ^a^
PG24(L)	0.78 ± 0.29	2.11 (1.79; 2.45)	0.66 ± 0.21 ^d^	0.90 (0.86; 0.99) ^c^

Note. Differences with the P-F(H) or P-F(L) group (^a^), between the PT4(H)/PT4(L) and PT16(H)/PT16(L) groups or between the PG4(H)/PG4(L) and PG16(H)/PG16(L) groups (^b^), between the PT16(H) and PT24(H) groups or between the PG16(L) and PG24(L) groups (^c^), and between the PT4(H)/PT4(L) and PT24(H)/PT24(L) groups or between the PG4(H)/PG4(L) and PG24(H)/PG24(L) groups (^d^) are significant at *p* < 0.05. The gene expression, quantified in Relative Quantification (RQ) values, is presented as mean ± SEM (normally distributed), except for *Egr1* gene expression in the PCOS(L) cohort and *Adamts1* and *Cyp19a1* gene expression in the PCOS(L) cohort, where their values are presented as median and interquartile range (25%; 75%) (non-normally distributed). In all groups: *n* = 4.

**Table 6:** The number of tertiary and preovulatory follicles, new-generation corpora lutea, and follicular cysts in the ovaries of PCOS rats treated with Follimag and Cetrotide, belonging to cohorts PCOS(H) and PCOS(L), as well as the effect of ovulation inducers, TP03 and hCG, on them at different time intervals (4, 16, and 24 h).

Group	Tertiary Follicles, Numbers	Preovulatory Follicles, Numbers	Corpora Lutea (New), Numbers	Follicular Cysts, Numbers
High-progesterone cohort, PCOS(H)				
P-F(H)	14.00 ± 2.42	5.25 ± 1.11	ND	5.00 ± 1.68
PT4(H)	5.75 ± 0.85 *	3.25 ± 0.63	ND	3.35 ± 1.31
PT16(H)	8.00 ± 1.58	1.75 ± 0.25 *	0.50 (0.00; 1.75)	3.50 ± 1.25
PT24(H)	11.25 ± 2.46	1.25 ± 0.95 *	3.00 (3.00; 3.75)	2.75 ± 1.18
PG4(H)	4.75 ± 0.25 *	3.50 ± 0.65	ND	4.00 ± 1.08
PG16(H)	7.50 ± 1.89	2.75 ± 1.11	0.50 (0.00; 1.00)	4.00 ± 0.91
PG24(H)	11.75 ± 0.85	1.25 ± 0.95 *	4.50 (2.50; 5.00)	4.75 ± 1.65
Low-progesterone cohort, PCOS(L)				
P-F(L)	9.75 ± 1.03	4.00 (3.00; 5.75)	ND	5.25 ± 2.87
PT4(L)	7.50 ± 1.32	2.50 (1.25; 3.75)	ND	3.50 ± 0.29
PT16(L)	6.75 ± 1.49	0.50 (0.00; 1.00) *	2.00(1.25; 2.75)	3.75 ± 0.63
PT24(L)	9.75 ± 2.39	ND	3.00 (1.50; 3.75)	5.00 ± 0.58
PG4(L)	10.50 ± 2.33	2.50 (2.00; 3.00)	ND	3.25 ± 0.85
PG16(L)	8.50 ± 2.33	0.50 (0.00; 1.75) *	1.50 (0.25; 3.50)	3.50 ± 0.65
PG24(L)	10.75 ± 3.09	0.50 (0.00; 1.00) *	2.50(0.50; 3.75)	5.75 ± 1.97

Note. *—differences with the P-F(H) or P-F(L) group are significant at *p* < 0.05. The number of tertiary follicles and follicular cysts, as well as PFs in the groups from the PCOS(H) cohort, are presented as mean ± SEM (with normal distribution), while the number of PFs in the groups from the PCOS(L) cohort and the number of corpora lutea are presented as the median and interquartile range (25%; 75%) (with non-normal distribution). In all groups: *n* = 4. ND—corpora lutea or PFs are not detected.

**Table 7:** Body and ovarian weights, hormone levels, ovarian gene expression, and ovarian morphological analysis data in PCOS female rats with high and low blood progesterone levels.

Indicator	Initially High Progesterone	Differences (Yes/No)	Initially Low Progesterone
Body weight	Norm	Yes *	Norm
Ovarian weight	Increased	No	Norm
Ovarian weight/body weight	Increased	No	Increased
Estrous cycle	Irregular, predominance of metestrus	Yes	Irregular, predominance of diestrus and proestrus
Progesterone	Significantly increased	Yes */Yes	Norm
Estradiol	Increased	Yes */Yes	Norm
Testosterone	Increased	No	Increased
LH	Decreased	No	Decreased
Glucose_0,120_, AUC_0–120_	Norm	No	Norm
Insulin_0_, IR_0_ index	Norm	No	Norm
Insulin_120_, IR_120_ index	Increased	No	Norm
Leptin_0,120_	Norm	No	Norm
*Lhcgr*	Increased	No	Increased
*Fshr*	Increased	Yes	Norm
*Star*	Norm	Yes	Decreased
*Cyp11a1*	Norm	Yes	Decreased
*Adamts1*	Norm	Yes	Decreased
*InsR*	Increased	No	Norm
Number of tertiary follicles	Decreased	No	Decreased
Number of PFs	Norm	No	Norm
Number of corpora lutea **	Norm	No	Decreased
Number of follicular cysts	Present in significant quantities	No	Present in significant quantities

Note. *—differences when comparing the combined groups in PCOS(H) and PCOS(L) cohorts (see Figure 2); **—corpora lutea from the previous cycle (correspond to the diestrus/proestrus phases).

## Data Availability

The original contributions presented in this study are included in the article/Supplementary Materials. Further inquiries can be directed to the corresponding author.

## Supplementary Materials

The following supporting information can be downloaded at https://www.mdpi.com/article/10.3390/ijms27062748/s1.

## Abbreviations

The following abbreviations are used in this manuscript:

ART	assisted reproductive technologies
DHEA	dehydroepiandrosterone
GnRH	gonadotropin-releasing hormone
hCG	human chorionic gonadotropin
iGTT	intraperitoneal glucose tolerance test
IR index	insulin resistance index
LH	luteinizing hormone
LHR	luteinizing hormone receptor
OHSS	ovarian hyperstimulation syndrome
PCOS	polycystic ovary syndrome
PF	preovulatory follicles
RQ	relative quantification
TP03	5-amino-*N-tert*-butyl-2-(methylsulfanyl)-4-(3-(nicotinamido)phenyl)thieno[2,3-d]-pyrimidine-6-carboxamide (allosteric LHR agonist)

## References

[1] Teede H., Deeks A., Moran L. (2010). Polycystic ovary syndrome: A complex condition with psychological, reproductive and metabolic manifestations that impacts on health across the lifespan. BMC Med..

[2] Salari N., Nankali A., Ghanbari A., Jafarpour S., Ghasemi H., Dokaneheifard S., Mohammadi M. (2024). Global prevalence of polycystic ovary syndrome in women worldwide: A comprehensive systematic review and meta-analysis. Arch. Gynecol. Obstet..

[3] Neven A.C.H., Forslund M., Ranasinha S., Sethi P., Dhungana R.R., Mousa A., Tay C.T., Teede H., Boyle J.A. (2026). Prevalence of polycystic ovary syndrome: A global and regional systematic review and meta-analysis. Hum. Reprod. Update.

[4] Teede H.J., Tay C.T., Laven J., Dokras A., Moran L.J., Piltonen T.T., Costello M.F., Boivin J., Redman L.M., Boyle J.A. (2023). Recommendations from the 2023 International Evidence-based Guideline for the Assessment and Management of Polycystic Ovary Syndrome. Fertil. Steril..

[5] Stener-Victorin E., Teede H., Norman R.J., Legro R., Goodarzi M.O., Dokras A., Laven J., Hoeger K., Piltonen T.T. (2024). Polycystic ovary syndrome. Nat. Rev. Dis. Primers.

[6] Azziz R. (2018). Polycystic Ovary Syndrome. Obstet. Gynecol..

[7] Teede H.J., Misso M.L., Costello M.F., Dokras A., Laven J., Moran L., Piltonen T., Norman R.J., Andersen M., Azziz R. (2018). Recommendations from the international evidence-based guideline for the assessment and management of polycystic ovary syndrome. Fertil. Steril..

[8] Pea J., Bryan J., Wan C., Oldfield A.L., Ganga K., Carter F.E., Johnson L.M., Lujan M.E. (2024). Ultrasonographic criteria in the diagnosis of polycystic ovary syndrome: A systematic review and diagnostic meta-analysis. Hum. Reprod. Update.

[9] Ding H., Zhang J., Zhang F., Zhang S., Chen X., Liang W., Xie Q. (2021). Resistance to the Insulin and Elevated Level of Androgen: A Major Cause of Polycystic Ovary Syndrome. Front. Endocrinol..

[10] Bizuneh A.D., Joham A.E., Teede H., Mousa A., Earnest A., Hawley J.M., Smith L., Azziz R., Arlt W., Tay C.T. (2025). Evaluating the diagnostic accuracy of androgen measurement in polycystic ovary syndrome: A systematic review and diagnostic meta-analysis to inform evidence-based guidelines. Hum. Reprod. Update.

[11] Cussen L., McDonnell T., Miller C., McIlroy M., O’Reilly M.W. (2025). Polycystic Ovary Syndrome: Origins and Implications: Polycystic ovary syndrome: The impact of androgen excess on metabolic health. Reproduction.

[12] Ruth K.S., Day F.R., Tyrrell J., Thompson D.J., Wood A.R., Mahajan A., Beaumont R.N., Wittemans L., Martin S., Busch A.S. (2020). Using human genetics to understand the disease impacts of testosterone in men and women. Nat. Med..

[13] Concepción-Zavaleta M.J., Fuentes-Mendoza J.M., Zavaleta-Gutiérrez F.E., Arias-Cantor B.Y., Figueredo-Rueda M.V., Coronado-Arroyo J.C., Paz-Ibarra J. (2025). Adolescent hyperandrogenism: Diagnostic challenges and therapeutic approaches. World J. Pediatr..

[14] Paixão L., Ramos R.B., Lavarda A., Morsh D.M., Spritzer P.M. (2017). Animal models of hyperandrogenism and ovarian morphology changes as features of polycystic ovary syndrome: A systematic review. Reprod. Biol. Endocrinol..

[15] Stener-Victorin E., Padmanabhan V., Walters K.A., Campbell R.E., Benrick A., Giacobini P., Dumesic D.A., Abbott D.H. (2020). Animal Models to Understand the Etiology and Pathophysiology of Polycystic Ovary Syndrome. Endocr. Rev..

[16] Kim E.J., Jang M., Choi J.H., Park K.S., Cho I.H. (2018). An Improved Dehydroepiandrosterone-Induced Rat Model of Polycystic Ovary Syndrome (PCOS): Post-pubertal Improve PCOS’s Features. Front. Endocrinol..

[17] Gharanjik F., Shojaeifard M.B., Karbalaei N., Nemati M. (2022). The Effect of Hydroalcoholic Calendula Officinalis Extract on Androgen-Induced Polycystic Ovary Syndrome Model in Female Rat. BioMed Res. Int..

[18] Wen X., Wang L., Lv S. (2024). Follicular development and endometrial receptivity of different androgen phenotypes in women with polycystic ovary syndrome. Front. Endocrinol..

[19] Yusuf A.N.M., Amri M.F., Ugusman A., Hamid A.A., Wahab N.A., Mokhtar M.H. (2023). Hyperandrogenism and Its Possible Effects on Endometrial Receptivity: A Review. Int. J. Mol. Sci..

[20] Zhu X., Ye H., Fu Y. (2016). The Utrogestan and hMG protocol in patients with polycystic ovarian syndrome undergoing controlled ovarian hyperstimulation during IVF/ICSI treatments. Medicine.

[21] Tandulwadkar S., Gupta S., Singh A., Mishra S., Singhania S. (2023). Medroxyprogesterone Acetate versus Gonadotropin-Releasing Hormone Antagonist for the Prevention of Premature Luteinizing Hormone Surge in hyper-responder women undergoing controlled ovarian stimulation for IVF/ICSI Cycles. JBRA Assist. Reprod..

[22] Massin N. (2017). New stimulation regimens: Endogenous and exogenous progesterone use to block the LH surge during ovarian stimulation for IVF. Hum. Reprod. Update.

[23] Nataraja S.G., Yu H.N., Palmer S.S. (2015). Discovery and Development of Small Molecule Allosteric Modulators of Glycoprotein Hormone Receptors. Front. Endocrinol..

[24] Lazzaretti C., Simoni M., Casarini L., Paradiso E. (2023). Allosteric modulation of gonadotropin receptors. Front. Endocrinol..

[25] Shpakov A.O. (2024). Hormonal and Allosteric Regulation of the Luteinizing Hormone/Chorionic Gonadotropin Receptor. Front. Biosci. (Landmark Ed.).

[26] van Koppen C.J., Zaman G.J., Timmers C.M., Kelder J., Mosselman S., van de Lagemaat R., Smit M.J., Hanssen R.G.J.M. (2008). A signaling-selective, nanomolar potent allosteric low molecular weight agonist for the human luteinizing hormone receptor. Naunyn-Schmied. Arch. Pharmacol..

[27] Bakhtyukov A.A., Derkach K.V., Gureev M.A., Dar’in D.V., Sorokoumov V.N., Romanova I.V., Morina I.Y., Stepochkina A.M., Shpakov A.O. (2020). Comparative Study of the Steroidogenic Effects of Human Chorionic Gonadotropin and Thieno[2,3-D]pyrimidine-Based Allosteric Agonist of Luteinizing Hormone Receptor in Young Adult, Aging and Diabetic Male Rats. Int. J. Mol. Sci..

[28] Bakhtyukov A.A., Derkach K.V., Sorokoumov V.N., Stepochkina A.M., Romanova I.V., Morina I.Y., Zakharova I.O., Bayunova L.V., Shpakov A.O. (2021). The Effects of Separate and Combined Treatment of Male Rats with Type 2 Diabetes with Metformin and Orthosteric and Allosteric Agonists of Luteinizing Hormone Receptor on Steroidogenesis and Spermatogenesis. Int. J. Mol. Sci..

[29] van de Lagemaat R., Raafs B.C., van Koppen C., Timmers C.M., Mulders S.M., Hanssen R.G. (2011). Prevention of the onset of ovarian hyperstimulation syndrome (OHSS) in the rat after ovulation induction with a low molecular weight agonist of the LH receptor compared with hCG and rec-LH. Endocrinology.

[30] Derkach K.V., Lebedev I.A., Morina I.Y., Bakhtyukov A.A., Pechalnova A.S., Sorokoumov V.N., Kuznetsova V.S., Romanova I.V., Shpakov A.O. (2023). Comparison of Steroidogenic and Ovulation-Inducing Effects of Orthosteric and Allosteric Agonists of Luteinizing Hormone/Chorionic Gonadotropin Receptor in Immature Female Rats. Int. J. Mol. Sci..

[31] Gerrits M., Mannaerts B., Kramer H., Addo S., Hanssen R. (2013). First evidence of ovulation induced by oral LH agonists in healthy female volunteers of reproductive age. J. Clin. Endocrinol. Metab..

[32] Poojary P.S., Nayak G., Panchanan G., Rao A., Kundapur S.D., Kalthur S.G., Mutalik S., Adiga S.K., Zhao Y., Bakkum-Gamez J. (2022). Distinctions in PCOS Induced by Letrozole Vs Dehydroepiandrosterone with High-fat Diet in Mouse Model. Endocrinology.

[33] Ikeda K., Baba T., Morishita M., Honnma H., Endo T., Kiya T., Saito T. (2014). Long-term treatment with dehydroepiandrosterone may lead to follicular atresia through interaction with anti-Mullerian hormone. J. Ovarian Res..

[34] Chen M.J., Chou C.H., Chen S.U., Yang W.S., Yang Y.S., Ho H.N. (2015). The effect of androgens on ovarian follicle maturation: Dihydrotestosterone suppress FSH-stimulated granulosa cell proliferation by upregulating PPARγ-dependent PTEN expression. Sci. Rep..

[35] Seow K.M., Ting C.H., Huang S.W., Ho L.T., Juan C.C. (2018). The use of dehydroepiandrosterone-treated rats is not a good animal model for the study of metabolic abnormalities in polycystic ovary syndrome. Taiwan. J. Obstet. Gynecol..

[36] Zhou D.N., Li S.J., Ding J.L., Yin T.L., Yang J., Ye H. (2018). MIF May Participate in Pathogenesis of Polycystic Ovary Syndrome in Rats through MAPK Signalling Pathway. Curr. Med. Sci..

[37] Olaniyan O.T., Femi A., Iliya G., Ayobami D., Godam E., Olugbenga E., Bamidele O., Chand Mali P. (2019). Vitamin C suppresses ovarian pathophysiology in experimental polycystic ovarian syndrome. Pathophysiology.

[38] Pechalnova A.S., Derkach K.V., Morina I.Y., Zorina I.I., Bayunova L.V., Romanova I.V., Chernenko E.E., Shpakov A.O. (2025). A Comparative Study of Dehydroepiandrosterone-Induced Polycystic Ovary Syndrome Models in Immature and Prepubertal Female Rats. J. Evol. Biochem. Physiol..

[39] Joshi A. (2024). PCOS stratification for precision diagnostics and treatment. Front. Cell Dev. Biol..

[40] Joham A.E., Piltonen T., Lujan M.E., Kiconco S., Tay C.T. (2022). Challenges in diagnosis and understanding of natural history of polycystic ovary syndrome. Clin. Endocrinol..

[41] Phy J.L., Conover C.A., Abbott D.H., Zschunke M.A., Walker D.L., Session D.R., Tummon I.S., Thornhill A.R., Lesnick T.G., Dumesic D.A. (2004). Insulin and messenger ribonucleic acid expression of insulin receptor isoforms in ovarian follicles from nonhirsute ovulatory women and polycystic ovary syndrome patients. J. Clin. Endocrinol. Metab..

[42] Liu N., Ma Y., Wang S., Zhang X., Zhang Q., Zhang X., Fu L., Qiao J. (2012). Association of the genetic variants of luteinizing hormone, luteinizing hormone receptor and polycystic ovary syndrome. Reprod. Biol. Endocrinol..

[43] Dumesic D.A., Padmanabhan V., Chazenbalk G.D., Abbott D.H. (2022). Polycystic ovary syndrome as a plausible evolutionary outcome of metabolic adaptation. Reprod. Biol. Endocrinol..

[44] Prosperi S., Chiarelli F. (2025). Insulin resistance, metabolic syndrome and polycystic ovaries: An intriguing conundrum. Front. Endocrinol..

[45] Upadhyay S., Mazumder A., Das S. (2025). Unraveling the Complexity of Polycystic Ovary Syndrome: Biomarkers for Diagnosis, Prognosis, and Treatment. Curr. Pharm. Des..

[46] Jancova P., Ismail K., Vistejnova L. (2025). Relationship between MASLD and women’s health: A review. Women’s Health.

[47] Apter D., Bützow T., Laughlin G.A., Yen S.S. (1994). Accelerated 24-hour luteinizing hormone pulsatile activity in adolescent girls with ovarian hyperandrogenism: Relevance to the developmental phase of polycystic ovarian syndrome. J. Clin. Endocrinol. Metab..

[48] García-Rudaz M.C., Ropelato M.G., Escobar M.E., Veldhuis J.D., Barontini M. (1998). Augmented frequency and mass of LH discharged per burst are accompanied by marked disorderliness of LH secretion in adolescents with polycystic ovary syndrome. Eur. J. Endocrinol..

[49] Coyle C., Campbell R.E. (2019). Pathological pulses in PCOS. Mol. Cell. Endocrinol..

[50] McCartney C.R., Campbell R.E., Marshall J.C., Moenter S.M. (2022). The role of gonadotropin-releasing hormone neurons in polycystic ovary syndrome. J. Neuroendocrinol..

[51] Dumesic D.A., Abbott D.H., Eisner J.R., Goy R.W. (1997). Prenatal exposure of female rhesus monkeys to testosterone propionate increases serum luteinizing hormone levels in adulthood. Fertil. Steril..

[52] Foecking E.M., Szabo M., Schwartz N.B., Levine J.E. (2005). Neuroendocrine consequences of prenatal androgen exposure in the female rat: Absence of luteinizing hormone surges, suppression of progesterone receptor gene expression, and acceleration of the gonadotropin-releasing hormone pulse generator. Biol. Reprod..

[53] Abbott D.H., Nicol L.E., Levine J.E., Xu N., Goodarzi M.O., Dumesic D.A. (2013). Nonhuman primate models of polycystic ovary syndrome. Mol. Cell. Endocrinol..

[54] McNeilly A.S., Duncan W.C. (2013). Rodent models of polycystic ovary syndrome. Mol. Cell. Endocrinol..

[55] Abramovich D., Irusta G., Bas D., Cataldi N.I., Parborell F., Tesone M. (2012). Angiopoietins/TIE2 system and VEGF are involved in ovarian function in a DHEA rat model of polycystic ovary syndrome. Endocrinology.

[56] Bakhtyukov A.A., Derkach K.V., Fokina E.A., Lebedev I.A., Sorokoumov V.N., Bayunova L.V., Shpakov A.O. (2023). Effect of Different Luteinizing Hormone Receptor Agonists on Ovarian Steroidogenesis in Mature Female Rats. J. Evol. Biochem. Physiol..

[57] Derkach K.V., Morina I.Y., Pechalnova A.S., Bayunova L.V., Zorina I.I., Ryzhova M.A., Romanova I.V., Shpakov A.O. (2025). Stimulation of ovarian steroidogenesis and ovulation in mature female rats with type 2 diabetes mellitus. J. Evol. Biochem. Physiol..

[58] Newton C.L., Whay A.M., McArdle C.A., Zhang M., van Koppen C.J., van de Lagemaat R., Segaloff D.L., Millar R.P. (2011). Rescue of expression and signaling of human luteinizing hormone G protein-coupled receptor mutants with an allosterically binding small-molecule agonist. Proc. Natl. Acad. Sci. USA.

[59] Deswal R., Nanda S., Dang A.S. (2019). Association of Luteinizing hormone and LH receptor gene polymorphism with susceptibility of Polycystic ovary syndrome. Syst. Biol. Reprod. Med..

[60] Lei C., Wang J., Li X., Mao Y.Y., Yan J.Q. (2023). Changes of insulin receptors in high fat and high glucose diet mice with insulin resistance. Adipocyte.

[61] Stepochkina A.M., Bakhtyukov A.A., Derkach K.V., Sorokoumov V.N., Shpakov A.O. (2022). A Comparative Study of the Steroidogenic Effect of 5-Amino-N-tert-butyl-2-(methylthio)-4-(3-(nicotinamido)phenyl)thieno[2,3-d]-pyrimidine-6-carboxamide and Chorionic Gonadotropin with Different Methods of Administration to Male Rats. J. Evol. Biochem. Physiol..

[62] Singh P., Srivastava R.K., Krishna A. (2016). Effects of gonadotropin-releasing hormone agonist and antagonist on ovarian activity in a mouse model for polycystic ovary. J. Steroid Biochem. Mol. Biol..

[63] Hossain M.M., Cao M., Wang Q., Kim J.Y., Schellander K., Tesfaye D., Tsang B.K. (2013). Altered expression of miRNAs in a dihydrotestosterone-induced rat PCOS model. J. Ovarian Res..

[64] Salehi R., Mazier H.L., Nivet A.L., Reunov A.A., Lima P., Wang Q., Fiocco A., Isidoro C., Tsang B.K. (2020). Ovarian mitochondrial dynamics and cell fate regulation in an androgen-induced rat model of polycystic ovarian syndrome. Sci. Rep..

[65] Hanssen R.G.J.M., Timmers C.M. (2003). Thieno[2,3-d]pyrimidines with Combined LH and FSH Agonistic Activity. Patent.

[66] He Y., Li X., Li Y., Kuai D., Zhang H., Wang Y., Tian W. (2024). Dehydroepiandrosterone with a high-fat diet treatment at inducing polycystic ovary syndrome in rat model. Steroids.

[67] Cora M.C., Kooistra L., Travlos G. (2015). Vaginal Cytology of the Laboratory Rat and Mouse: Review and Criteria for the Staging of the Estrous Cycle Using Stained Vaginal Smears. Toxicol. Pathol..

[68] Ajayi A.F., Akhigbe R.E. (2020). Staging of the estrous cycle and induction of estrus in experimental rodents: An update. Fertil. Res. Pract..

[69] Schmittgen T.D., Livak K.J. (2008). Analyzing real-time PCR data by the comparative C(T) method. Nat. Protoc..

[70] Melo M.A., Meseguer M., Garrido N., Bosch E., Pellicer A., Remohí J. (2006). The significance of premature luteinization in an oocyte-donation programme. Hum. Reprod..

[71] Venetis C.A., Kolibianakis E.M., Bosdou J.K., Tarlatzis B.C. (2013). Progesterone elevation and probability of pregnancy after IVF: A systematic review and meta-analysis of over 60,000 cycles. Hum. Reprod. Update.

