# Supplementary material for: The Stimulating Effect of Low-Molecular-Weight Luteinizing Hormone Receptor Agonist on Steroidogenesis and Ovulation in Female Rats with Dehydroepiandrosterone-Induced Polycystic Ovary Syndrome
Source: Int J Mol Sci. 2026 Mar 18;27(6):2748. doi: 10.3390/ijms27062748 (PMC13026792; doi:10.3390/ijms27062748)
Supplement: Supplementary file 1 [file ijms-27-02748-s001.zip › Figure S1 new.pdf]

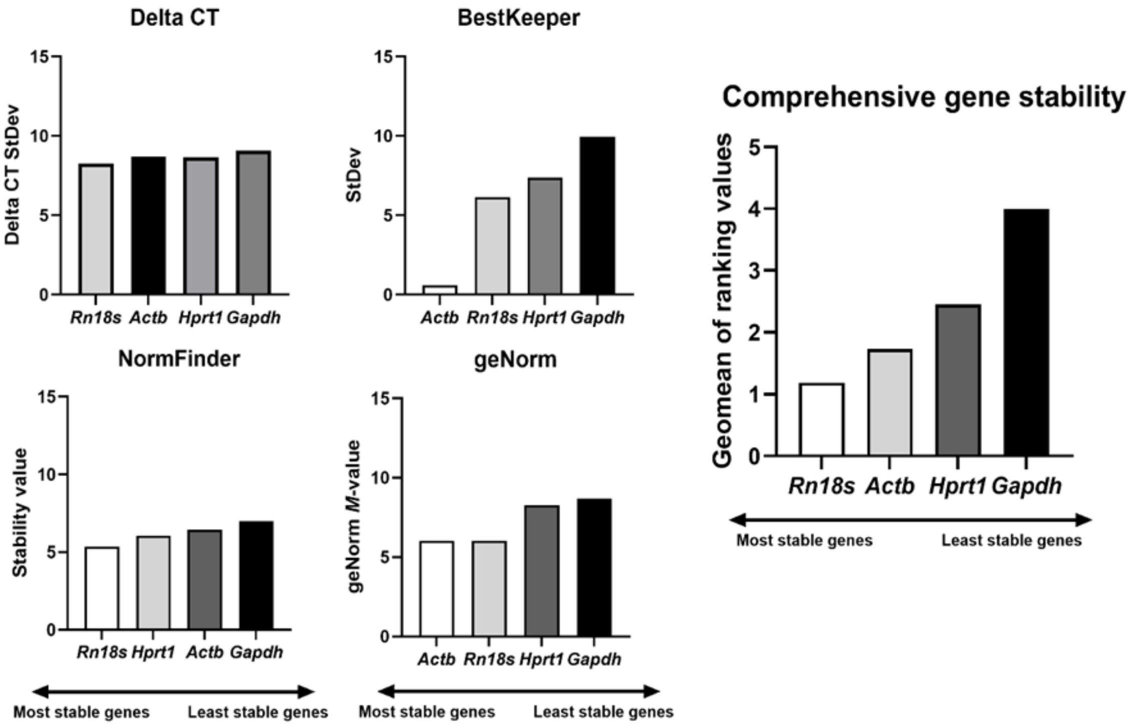

Figure S1. The reference gene stability rankings in ovaries of the control rats and animals with DHEA-induced PCOS treated with LHR-agonists at different time points. The gene expression stability was assessed by RefFinder online tool.
