# Supplementary material for: The Stimulating Effect of Low-Molecular-Weight Luteinizing Hormone Receptor Agonist on Steroidogenesis and Ovulation in Female Rats with Dehydroepiandrosterone-Induced Polycystic Ovary Syndrome
Source: Int J Mol Sci. 2026 Mar 18;27(6):2748. doi: 10.3390/ijms27062748 (PMC13026792; doi:10.3390/ijms27062748)
Supplement: Supplementary file 1 [file ijms-27-02748-s001.zip › Table S1.pdf]

Table S1. Blood progesterone levels in female rats with PCOS 48 hours after treatment with Follimag, before administration of LHCGR agonists

**Group**	**Progesterone, nmol/L**
P-F(H), *n*=4	46.6 ± 30.7
PT4(H), *n*=4	58.2 ± 43.7
PT16(H), *n*=4	35.3 ± 8.9
PT24(H), *n*=4	32.2 ± 15.4
PG4(H), *n*=4	16.6 ± 7.5
PG16(H), *n*=4	24.6 ± 5.3
PG24(H), *n*=4	54.1 ± 23.0
P-F(L), *n*=4	8.6 ± 2.1
PT4(L), *n*=4	6.9 ± 2.2
PT16(L), *n*=4	37.5 ± 24.8
PT24(L), *n*=4	9.9 ± 2.2
PG4(L), *n*=4	10.3 ± 1.6
PG16(L), *n*=4	15.4 ± 2.5
PG24(L), *n*=4	7.6 ± 1.6

Note. All experimental PCOS groups were formed based on a random independent sample of rats from the PCOS(H) and PCOS(L) cohorts. Data are presented as *mean ± SEM*.
