# Supplementary material for: The Stimulating Effect of Low-Molecular-Weight Luteinizing Hormone Receptor Agonist on Steroidogenesis and Ovulation in Female Rats with Dehydroepiandrosterone-Induced Polycystic Ovary Syndrome
Source: Int J Mol Sci. 2026 Mar 18;27(6):2748. doi: 10.3390/ijms27062748 (PMC13026792; doi:10.3390/ijms27062748)
Supplement: Supplementary file 1 [file ijms-27-02748-s001.zip › Table S2_new.pdf]

**Table S2.** Primers for studying the expression of target and housekeeping genes in the ovaries of female rats using RT-PCR

| Gene | Forward/Reverse Sequence | Product Size (bp) | Annealing Temperature (°C) | Genbank Accession Number |
|---|---|---|---|---|
| *Lhcgr* | (For) CTGCGCTGTCCTGGCC<br>(Rev) CGACCTCATTAAGTCCCCTGAA | 103 | 55 | NM_012978.1 |
| *Fshr* | (For) GGAACGCCATTGAACTGAGGT<br>(Rev) AGGTTGGAGAACACATCTGCC | 145 | 55 | NM_199237.2 |
| *Star* | (For) AAGGCTGGAAGAAGGAAAGC<br>(Rev) CACCTGGCACCACCTTACTT | 66 | 55 | NM_031558.3 |
| *Cyp11a1* | (For) TATTCCGCTTTGCCTTTGAG<br>(Rev) CACGATCTCCTCCAACATCC | 74 | 55 | NM_017286.3 |
| *Cyp19a1* | (For) GGTATCAGCCTGTCGTGGAC<br>(Rev) AGCCTGTGCATTCTTCCGAT | 118 | 56 | NM_017085.2 |
| *Adamts1* | (For) CTGCTGCCCTCAGGTGTAAA<br>(Rev) TGAGTGGACTAAAGCTGCGG | 187 | 55 | NM_024400.2 |
| *Egr1* | (For) CGTAATCCAAGGGGGTCCAG<br>(Rev) GTGTAAGCTCATCCGAGCGA | 196 | 55 | NM_012551.3 |
| *Cox2* | (For) ATCAAAGCCTTCGCCACTCA<br>(Rev) ACGGGGCCTTCAAAATGTCT | 79 | 55 | NM_017232.4 |
| *Vegfa* | (For) CACTGGACCCTGGCTTTACT<br>(Rev) GACGTCCATGAACTTCACCA | 62 | 55 | NM_001287114.1 |
| | Housekeeping genes | | | |
| *Actb* | (For) CTGGCACCACACCTTCTACA<br>(Rev) AGGTCTCAAACATGATCTGGGT | 125 | 55 | NM_031144.3 |
| *Rn18s* (18S rRNA) | (For) GGACACGGACAGGATTGACA<br>(Rev) ACCCACGGAATCGAGAAAGA | 50 | 56 | NR_046237.3 |
| *Hprt1* | (For) GCGAAAGTGGAAAAGCCAAGT<br>(Rev) GCCACATCAACAGGACTCTTGTA | 76 | 56 | NM_012583.2 |
| *Gapdh* | (For) GTGTTCCTACCCCCAATGTATCC<br>(Rev) GATGTCATCATACTTGGCAGGTTT | 74 | 56 | NM_001394060.2 |
